# A *forkhead* Transcription Factor Is Wound-Induced at the Planarian Midline and Required for Anterior Pole Regeneration

**DOI:** 10.1371/journal.pgen.1003999

**Published:** 2014-01-09

**Authors:** M. Lucila Scimone, Sylvain W. Lapan, Peter W. Reddien

**Affiliations:** Howard Hughes Medical Institute, MIT Biology and Whitehead Institute for Biomedical Research, Cambridge, Massachusetts, United States of America; University of Oxford, United Kingdom

## Abstract

Planarian regeneration requires positional information to specify the identity of tissues to be replaced as well as pluripotent neoblasts capable of differentiating into new cell types. We found that wounding elicits rapid expression of a gene encoding a Forkhead-family transcription factor, *FoxD*. Wound-induced *FoxD* expression is specific to the ventral midline, is regulated by Hedgehog signaling, and is neoblast-independent. *FoxD* is subsequently expressed within a medial subpopulation of neoblasts at wounds involving head regeneration. Ultimately, *FoxD* is co-expressed with multiple anterior markers at the anterior pole. Inhibition of *FoxD* with RNA interference (RNAi) results in the failure to specify neoblasts expressing anterior markers (*notum* and *prep*) and in anterior pole formation defects. *FoxD(RNAi)* animals fail to regenerate a new midline and to properly pattern the anterior blastema, consistent with a role for the anterior pole in organizing pattern of the regenerating head. Our results suggest that wound signaling activates a *forkhead* transcription factor at the midline and, if the head is absent, *FoxD* promotes specification of neoblasts at the prior midline for anterior pole regeneration.

## Author Summary

Regeneration is widespread in the animal kingdom. Planarians are able to regenerate entire bodies from almost any fragment type. This ability requires a cell population called neoblasts, which include pluripotent stem cells, for the production of all missing tissues, as well as the information to form and pattern correct new tissue types. Two discrete regions of the body, called poles, are found at the anterior and posterior ends of the animal. Here we investigate the role of a gene encoding a Forkhead-family transcription factor, *FoxD*, in formation of the anterior pole. *FoxD* is expressed at the anterior pole and following injury, *FoxD* expression is induced in a restricted midline region of the animal. Next, *FoxD* is expressed in a subset of neoblasts at the midline. Inhibition of *FoxD* with RNA interference results in defective anterior pole regeneration, and subsequent failure to regenerate an organized head pattern around a new midline. *FoxD* is specifically required for anterior regeneration. These results suggest that there is a regenerative connection between the midline and the anterior pole.

## Introduction

Planarians can regenerate from nearly any injury, but how missing tissues are recognized and replaced is poorly understood. The adult population of proliferating cells (neoblasts) in *Schmidtea mediterranea* includes pluripotent stem cells [1] and is responsible for new tissue production in regeneration. New cell production at wounds produces an outgrowth called a blastema, which will replace some of the missing tissues [2]. At the molecular level, injuries trigger a rapid wound response program that includes conserved immediate early genes and patterning factors required for normal regeneration [3]. Wnt and Hedgehog (Hh) signaling pathways instruct the regeneration of the anterior-posterior (AP) axis, whereas the Bmp signaling pathway controls the regeneration of the dorsal-ventral (DV) axis [4]–[13]. Multiple genes required for positional identity control during embryonic development of other organisms, such as Wnt and Bmp signaling ligands, display constitutive regionalized expression in adult planarians and also guide pattern maintenance during tissue turnover [14].

Two distinct regions composed of a small cluster of cells located at the anterior and posterior animal extremities are referred to here as the anterior and posterior planarian poles. The poles are found at the midline of the animal and are subjects of current intense study. The anterior pole expresses *notum*, a Wnt inhibitor required for head regeneration [8], whereas the posterior pole expresses *wnt1* [7], [14]. A number of genes encoding proteins predicted to be involved in signaling pathways that regulate planarian body plan patterning, and that display regional expression in planarian muscle cells have been described [15]. The genes that display these unique attributes will be referred to as position control genes (PCGs) for simplicity of discussion, but it is important to note that patterning phenotypes have not yet been described for many of these genes. PCGs expressed broadly in the planarian head include the candidate Wnt inhibitor, *sFRP-1*; candidate FGF inhibitors *nou darake* (*ndk*) and *ndl-4*; and a homeodomain transcription factor, *prep* [6], [7], [14]–[17]. PCGs expressed broadly in the posterior region of the animal include genes encoding additional Wnt ligands, *wnt11-1*, *wnt11-2*, and *wntP-2/wnt11-5* and the Wnt receptor *frizzled-4* [6], [7], [14], [15], [18]; Hox genes are also expressed in the posterior [14]. Several PCGs are expressed at the planarian poles, but have broader expression domains that extend beyond the cluster of *notum* ^+^ or *wnt1* ^+^ cells at the animal head and tail tips.

How the poles are formed and the role they have in organizing regeneration of an animal with a properly patterned body plan is poorly understood. Two genes encoding transcription factors of the TALE-class homeodomain family, *prep* and *pbx*, are required for regeneration of the expression domains of anterior PCGs and the anterior pole marker *notum* [17], [19], [20]. *pbx* [20] and a LIM-homeobox gene (*djislet*, [21]) are required for regeneration of expression domains of posterior PCGs and the posterior pole marker *wnt1*. *follistatin*, which encodes a secreted regulator of TGF-β proteins, is also expressed at the anterior pole and is required for normal head regeneration [22], but strong inhibition of this gene can also result in the absence of blastema formation indicating a broader role of this gene during regeneration [23].

Forkhead-box (*Fox*) genes are an evolutionary ancient family of winged-helix transcription factors involved in a wide variety of biological processes such as regulation of cell proliferation, growth, and differentiation [24]. During embryogenesis, *Fox* genes are involved in organogenesis and patterning of several tissues from all three germ layers [25]. Mutations in *Fox* genes have a profound impact in human disease causing a variety of phenotypes, from eye abnormalities to speech impediments [25]–[27]. Some members of the *Fox* gene family are expressed in restricted regions of embryos. In *Drosophila*, genes encoding Forkhead-family proteins, *fkh*, *sloppy paired 1* and *2*, and *crocodile*, are all expressed in the anterior region of the embryo, and are required for midline establishment as well as head patterning [28]–[31]. In amphioxus, *FoxQ* is expressed at the anterior pole during embryogenesis [32]. In *Xenopus*, the forkhead family gene, *XDF1* is expressed in Spemann's organizer and at later stages in the anterior neural region [33]. In planarians, few *Fox* genes have been described. In particular, *DjFoxD* is expressed in few cells at the anterior pole region of the planarian *D. japonica* [34] and *FoxD* influences expression of *follistatin* in planarian heads [22], [34]. Given the potential importance of the planarian anterior pole in organizing head regeneration, we investigated the role of *Schmidtea mediterranea FoxD* in regeneration.

## Results

### Co-expression of *FoxD* with a number of anterior-expressed genes defines the anterior pole

**Figure 1:**
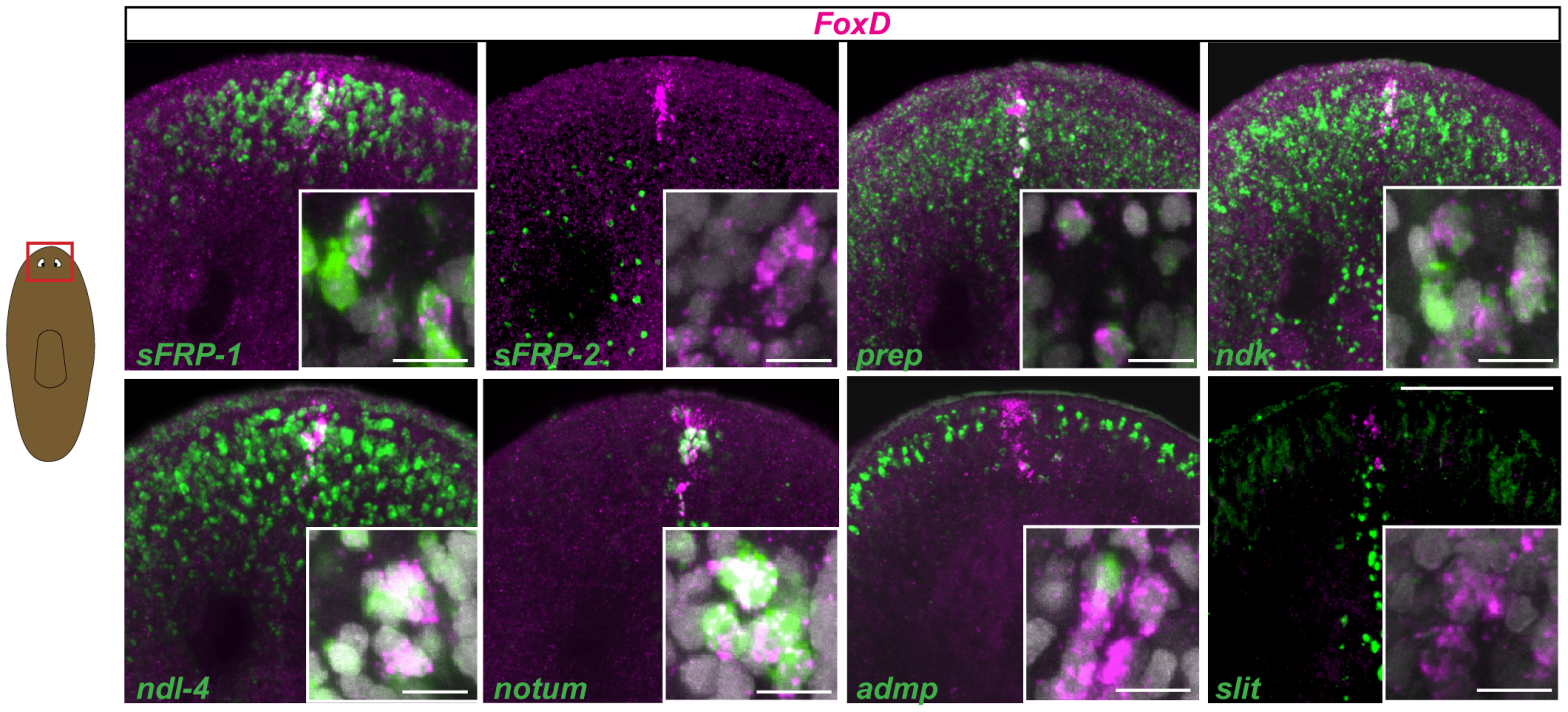
*FoxD* is expressed with multiple other PCGs at the anterior pole. Wild-type intact animals were labeled in double whole-mount fluorescence *in situ* hybridization (FISH) with *FoxD* (magenta) and several anterior and midline patterning genes (green). Percentages (mean ± SD) of *FoxD* cells co-expressing anterior markers are: 56±5% with *sFRP1*; 66±9% with *ndk*, 43±9% with *ndl-4*, 72±19% with *prep*, and 68±11% with *notum*; co-expression with the midline genes *admp* is 2±4%, and *slit* is 5±8% (n>50 *FoxD^+^* cells examined in each case). Red box in cartoon on the left shows the area depicted. Animals are anterior up; dorsal view. Insets show higher magnification images of co-expressing cells. Images shown are maximal intensity projections. Images are representative of results seen in >6 animals per panel. Scale bars for all panels, 100 µm. Inset scale bars, 10 µm.

A number of genes have been identified that are expressed in different domains of planarian heads. To provide a molecular definition of the anterior-most end of the planarian head, the anterior pole, we investigated the expression of a number of genes expressed near the planarian head tip. *FoxD* is expressed in a very small number of cells at the head tip (Fig. 1 and [22], [34]), but the pole and its role(s) are poorly defined; we focused our investigation of the anterior pole on the *Schmidtea mediterranea* ortholog of *DjFoxD*, *Smed-FoxD*, or *FoxD* in short (Fig. S1). *FoxD* expression in intact animals was dorsal-biased and most *FoxD^+^* cells also expressed the gene *notum*. *notum* is required for the head-versus-tail regeneration decision known as AP regeneration polarity, and encodes a secreted inhibitor of Wnt signaling [8]. Like *FoxD*, *notum* expression in uninjured animals is largely restricted to a very small number of cells at the head tip (Fig. 1 and [8]). *FoxD^+^* cells at the head tip also expressed multiple anterior markers (PCGs) that are expressed in AP transcriptional domains extending beyond the *FoxD^+^* cells, including *sFRP-1*, *ndk*, *ndl-4*, and *prep*, but not *sFRP-2* (Fig. 1). We also assessed whether *FoxD* is co-expressed with two planarian genes expressed at the DV boundary (a lateral domain surrounding the animal at the midpoint of dorsal and ventral surfaces) and/or at the midline (the median plane about which bilateral symmetry is organized): *admp*, encoding a BMP-family signaling ligand expressed in the ventral midline and at the DV boundary [10], [11] and *slit*, a conserved midline cue with a prominent role in regulation of axon guidance [35] expressed in the ventral and dorsal planarian midline [35]. *admp* and *slit* expression did not substantially coincide with pole cells expressing *FoxD*; midline *slit* expression did not reach the anterior-most region where *FoxD*-expressing cells are found (Fig. 1). We propose a definition for the planarian anterior pole in the mature head as the few cells restricted to the head tip and that co-express the highly restricted *FoxD* and *notum* genes together with a set of anterior PCGs, but displaying little expression of DV boundary and midline genes. The cellular basis for formation of these cells and the roles of these cells in regeneration are investigated below.

### *FoxD* expression is wound-induced in the ventral midline

**Figure 2:**
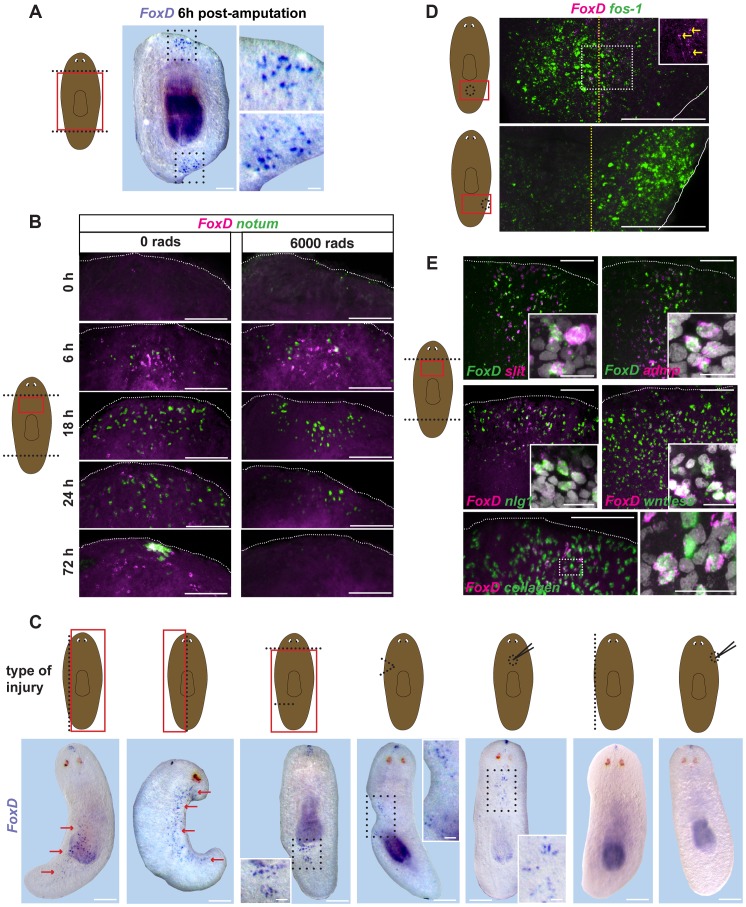
*FoxD* is wound-induced in the midline. (**A**) Wild-type animals were transversely amputated, fixed at six hours following wounding, and *FoxD* (purple) expression was analyzed by *in situ* hybridization. Red box in cartoon on the left shows the area imaged. All animals showed anterior and posterior *FoxD* expression at the midline domain following wounding (n>10). Animals are anterior up; ventral view. Area within dotted black box is enlarged in right panels. Scale bar, 500 µm. Right panels, scale bar, 100 µm. (**B**) Double FISH, *notum* (green) and *FoxD* (magenta) in wild-type (0 rads) and lethally irradiated animals (6,000 rads) at different time points following transverse amputation. Red box in cartoon on the left shows the area depicted. Images shown are maximal intensity projections. Images are representative of results seen in n>6 animals per panel. Dorsal view for the 72 hours time point, ventral view for all others. Dotted white line marks the approximate wound boundary. Scale bars, 100 µm. (**C**) Cartoons show injury types performed in wild-type animals. Dotted black line shows the wound site. Type of wound from left to right: parasagittal, sagittal, incision, wedge, dorsal midline puncture, lateral edge, and lateral puncture. Red box shows the region imaged below. Whole-mount *in situ* hybridization using *FoxD* (purple) RNA probe at six hours following wounding of wild-type animals. Red arrows indicate *FoxD* expression. Dotted black box is enlarged in the inset. Images are representative of results seen in >10 animals per wound type. Animals are anterior up, ventral view. Scale bars, 500 µm. Inset scale bars, 100 µm. (**D**) Double FISH, *FoxD* (magenta) and the immediate early gene *fos-1* (green) of wounded wild-type animals fixed at six hours following midline (upper panel) and lateral (lower panel) puncture injuries. Cartoon on the left shows type of wound, red box is area imaged. Dotted yellow line depicts the approximate midline, white line depicts the animal edge. Area within dotted white box is shown in the inset for *FoxD* expression. Yellow arrows point to *FoxD*-expressing cells at the midline. Images shown are maximal intensity projections. Images are representative of results seen in >10 animals per panel. Anterior is up, ventral view. Scale bars, 100 µm (**E**) Double FISH, *FoxD* (green for upper two panels, magenta all other panels) and midline, W2 genes or the muscle gene *collagen* of wild-type animals fixed six hours following transverse amputation. Percentage (mean ± SD) of *FoxD* co-expression with *slit* was 86.3±5.5% (n = 124 *FoxD^+^* cells examined), with collagen was 92.6±4.6% (n = 52 *FoxD^+^* cells examined). Red box shows the area imaged. Anterior wounds are up, ventral view. Insets show higher magnification of co-expressing cells, scale bars are 10 µm. For the double *FoxD*, *collagen* FISH, area within dotted white box is shown in the right panel. Images shown are maximal intensity projections. Images are representative of results seen in >5 animals per panel. Dotted white line marks the approximate wound boundary. Scale bars, 100 µm.

*FoxD* expression was highly induced by three hours following wounding in subepidermal cells, with expression peaking at approximately six hours after wounding (Fig. 2A). *FoxD* expression after amputation occurred at both anterior- and posterior-facing wounds, raising the possibility that *FoxD* is a generically wound-induced gene (Fig. 2A). *FoxD* expression after amputation was greatly diminished by 18 to 24 hours following injury (Fig. 2B), but increased again between 24 and 48 hours after amputation. At this later time *FoxD* expression was only observed at anterior-facing wounds that required the formation of a new anterior pole (Fig. 2B and Fig. S2A). Irradiation eliminates neoblasts [36], which comprise the entire population of dividing adult planarian cells; therefore, amputation experiments in irradiated animals can determine whether new gene expression in regeneration occurs in pre-existing cells at wound sites, or requires new cell production. Early wound-induced expression of *FoxD* was irradiation-insensitive, indicating that it is a transcriptional response in cells present at the time of wounding (Fig. 2B).

The large numbers of planarian wound-induced genes described so far are expressed broadly at the wound site [3], [8], [18], [37]. By contrast, wound-induced *FoxD* expression following amputation was unique: restricted to cells found in the ventral midline (Fig. 2A,B). To better understand the regulation of *FoxD* expression by wounding, we examined the impacts of several injury types on *FoxD* expression (Fig. 2C). First, we observed that expression of *FoxD* was not exclusive to wounds requiring pole regeneration; both parasagittal and sagittal amputations induced *FoxD* expression in the midline broadly along the wound site (first two cuts from the left, Fig. 2C). Incision into the planarian side with a scalpel, a wound not requiring blastema formation for repair, was sufficient to induce *FoxD* expression. Strikingly, *FoxD* was expressed at anterior and posterior areas of the incised wound site, but only within the midline on the ventral side (third cut from left, Fig. 2C). This wound-induced *FoxD* expression occurred even in the absence of the anterior pole, indicating that local cues rather than signals from the pole control wound-induced expression of *FoxD*. An even more minor injury, a dorsal puncture with a needle (third panel from right, Fig. 2C), induced *FoxD* expression in the vicinity of the dorsal puncture, but only ventrally in the midline. All of these wound types impinged upon the midline of the animal, raising the possibility that any midline injury is sufficient to trigger *FoxD* expression, regardless of whether blastema formation would be required for repair or not. Supporting this hypothesis, two wound types that do not damage the midline (lateral puncture and lateral edge removal; first and second panels from right, Fig. 2C) did not trigger expression of *FoxD*. These two wounds did, by contrast, elicit normal expression of other wound-induced genes (Fig. 2D).

The wound-induced expression of *FoxD* occurred within ventral midline cells expressing the midline markers *admp* and *slit* (Fig. 2E). Furthermore, *FoxD* was also co-expressed at wounds with other defined wound-induced genes, such as *noggin-like1* (*nlg1*) [3], [38], *wntless* [3], [12], [38], *notum* [8], and *wnt1* [37] (Fig. 2E and Fig. S2B). Wound-induced genes that peak in expression around six hours following injury and that are mostly expressed subepidermally at wound sites are known as W2 genes [3]. Although most W2 genes are predicted to be secreted proteins, such as signaling and matrix remodeling factors, our results indicate that the transcription factor *FoxD* itself belongs in this category. W2 gene expression is induced in muscle cells expressing *collagen* [15], and most wound-induced *FoxD* expressing cells (92.5±4.6%) also co-expressed the muscle gene *collagen* (Fig. 2E). We conclude that *FoxD* is a wound-induced gene in planarian muscle, but unique among known planarian wound-induced genes with expression occurring only in ventral midline cells and only following injury that impinges on the midline. *FoxD* also defines a fourth gene (together with *wnt1*, *notum*, and *follistatin*) that is wound-induced and subsequently expressed in either the anterior or posterior pole.

### *FoxD* is expressed in a subset of *smedwi-1* ^+^ cells during anterior pole regeneration

**Figure 3:**
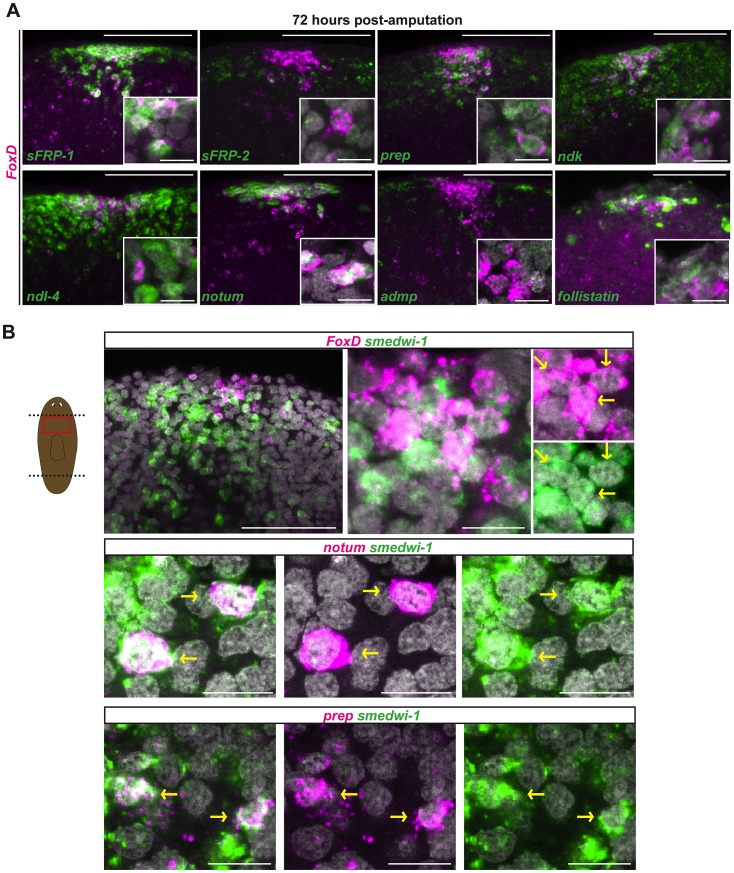
*FoxD* is expressed in anterior pole progenitors. Double FISH in wild-type head blastemas 72 hours after transverse amputation. Red box in cartoon shows the region imaged. Images shown are maximal intensity projections. Images are representative of n>5 animals per panel. Anterior is up, dorsal view. (**A**) *FoxD* (magenta) and PCGs (green). Percentages (mean ± SD) of *FoxD* cells co-expressing anterior patterning genes and anterior pole markers are as follows: 95±2% with *sFRP1*; 93±3% with *ndk*; 81±9% with *ndl-4*; 86±5% with *prep*; and 74±11% with *notum*; co-expression with the midline gene *admp* is 3±5% (n>120 *FoxD^+^* cells examined in each case). Scale bars, 100 µm. Inset shows higher magnification of co-expressing cells, scale bars are 10 µm. (**B**) *FoxD*, *notum*, and *prep* (magenta), *smedwi-1* (green). Percentage (mean ± SD) of *FoxD* cells co-expressing *smedwi-1* was 39.7±13.3% (n = 157 *FoxD^+^* cells examined); percentage of *notum* cells co-expressing *smedwi-1* was 22.9±6.1% (n = 111 *notum^+^* cells examined). Yellow arrows point to double-labeled cells. Scale bars in upper left panel, 100 µm; other panels, 10 µm.

The second phase of *FoxD* expression during regeneration, initiating around 24 hours post-amputation, occurred in cells that were coalesced at the anterior pole. This expression phase presented the opportunity to define the cellular steps of anterior pole formation. Irradiated animals did not form an anterior pole and did not express *FoxD* at 24 to 72 hours following wounding (Fig. 2B), indicating the requirement of neoblasts for this process. *FoxD*-expressing cells at the regenerating anterior pole at this time also co-expressed multiple anterior PCGs as well as the anterior pole-expressing gene *notum* (Fig. 3A). *follistatin*, which is required for anterior regeneration [22], [23], was also co-expressed with *FoxD* at the regenerating anterior pole at this regeneration stage (Fig. 3A). *FoxD* expression gradually became restricted with time to fewer cells at the regenerating anterior pole, adopting the same appearance as in intact animals (Figs. 1 and 3A).

The planarian *smedwi-1* gene encodes a PIWI family protein and is expressed in all dividing adult planarian cells, marking the neoblast population [1]. We found that some *FoxD*-expressing cells located at the forming anterior pole co-expressed *smedwi-1* in regenerating blastemas 72 hours following amputation (Fig. 3B). In addition to *FoxD*, expression of the anterior pole gene *notum* and the anterior-patterning gene *prep* was also found in *smedwi-1*-expressing neoblasts at the regenerating anterior pole (Fig. 3B). These data suggest that by three days of regeneration some neoblasts have been specified to produce the new pole cells of the regenerating head, potentially marking the first cellular step in formation of a new anterior pole.

### Hh signaling impacts wound-induced *FoxD* expression

**Figure 4:**
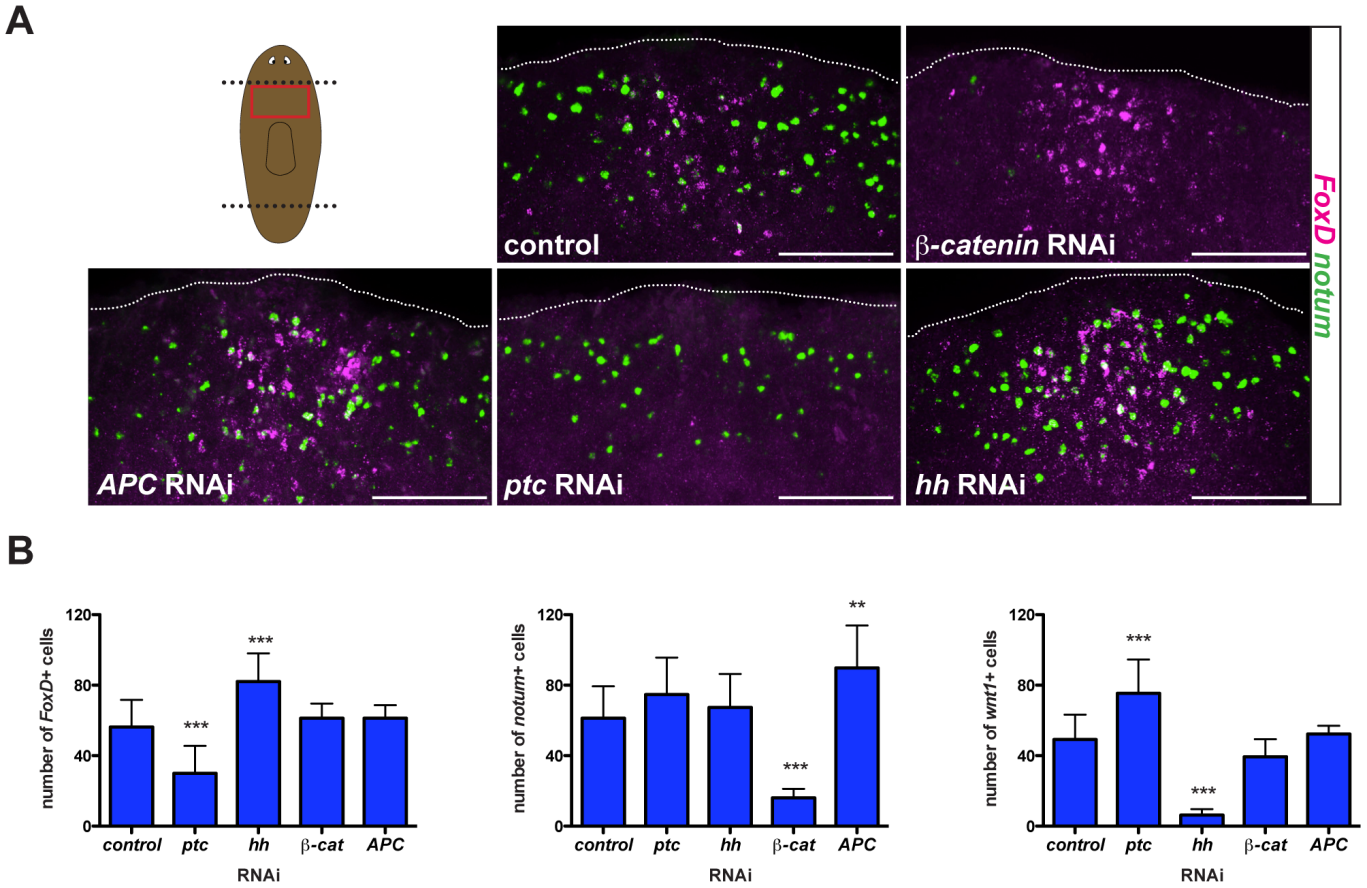
Hh signaling is required for wound-induced *FoxD* expression. (**A**) Double FISH; *notum* (green) and *FoxD* (magenta) of RNAi fed animals fixed six hours following transverse amputation. Red box in cartoon shows region imaged. Images shown are maximal intensity projections. Dotted white line marks the approximate wound boundary. Anterior wounds are up, ventral view. Scale bars for all panels, 100 µm. (**B**) Graphs show number of cells expressing *FoxD, notum*, or *wnt1* in the different RNAi conditions. Data are shown as mean ± SD, and analyzed using a one-way ANOVA test; **p<0.01, ***p<0.001, n>10 animals per RNAi condition.

Both Wnt and Hh signaling pathways are required for the head-versus-tail regeneration decision made at planarian amputation planes [4]–[8], [39]. To test whether wound-induced *FoxD* expression was regulated by these signaling pathways, we inhibited Wnt (*β-catenin* and *APC*) and Hh (*patched* (*ptc*) and *hedgehog* (*hh*)) pathway genes with RNAi and examined *FoxD* expression six hours following amputation. As controls, we analyzed the numbers of *notum*- and *wnt1*-expressing cells in the different RNAi conditions and, as expected from prior reports [5], [8], *notum* expression was exclusively affected in *β-catenin* and *APC* RNAi animals and *wnt1* expression was affected following perturbation of the Hh signaling pathway (Figs. 4 and S3A). Perturbation of Wnt signaling did not affect wound-induced *FoxD* expression (Figs. 4 and S3A). By contrast, *ptc(RNAi)* animals displayed fewer than normal *FoxD*-expressing cells at wounds and *hh(RNAi)* animals had increased numbers of *FoxD*-expressing cells (Figs. 4 and S3A). *patched* (*ptc*) encodes a receptor for Hh that antagonizes pathway output [40]. Hh signaling therefore negatively regulates *FoxD* induction at the midline following wounding. Because *hh* impacts wound-induced *wnt1* expression oppositely to *FoxD* [4], [5], this result does not reflect a generic requirement for *hh* in wound-induced gene activation. *FoxD* expression at the anterior pole was normal in *hh(RNAi)* anterior blastemas (Fig. S3B), indicating a specific role for *hh* in regulating the *FoxD* wound-induced phase of expression.

*ptc(RNAi)* animals with a strong phenotype regenerate tails in place of heads, but we found no evidence that *FoxD* influences the head-versus-tail regeneration choice.

However, *ptc(RNAi)* animals with a weak phenotype (e.g., cyclopic heads) do resemble *FoxD(RNAi)* animals [4], [5]. Cyclopic or headless *ptc(RNAi)* animals showed decreased expression of the anterior PCG *sFRP-1* ([5] and Fig. S3D) and decreased expression of the anterior pole marker *notum* (Fig. S3D), indicating a defect in anterior pole regeneration. Therefore, the defect in *FoxD* expression might contribute to the *ptc(RNAi)* phenotype. In vertebrates, Sonic hedgehog (a member of the Hh family) signaling is required for normal forebrain development and midline induction [40], [41]. In planarians, *hh* is expressed ventrally and medially [4], [5]; the impact on wound-induced expression of *FoxD* at the midline raises the interesting possibility that *hh* might also have a role in midline biology in planarians. In *ptc(RNAi)* animals, midline expression of *slit* at six hours following amputation was normal (Fig. S3C). 86.3±5.5% of *FoxD*-expressing cells following wounding co-express the midline gene *slit* in wild-type animals. Therefore, these results indicate that the reduced wound-induced expression of *FoxD* in *ptc(RNAi)* animals is not a consequence of the absence of the midline cells that normally express *FoxD*.

### Disruption of pole formation with *FoxD* RNAi perturbs anterior regeneration

**Figure 5:**
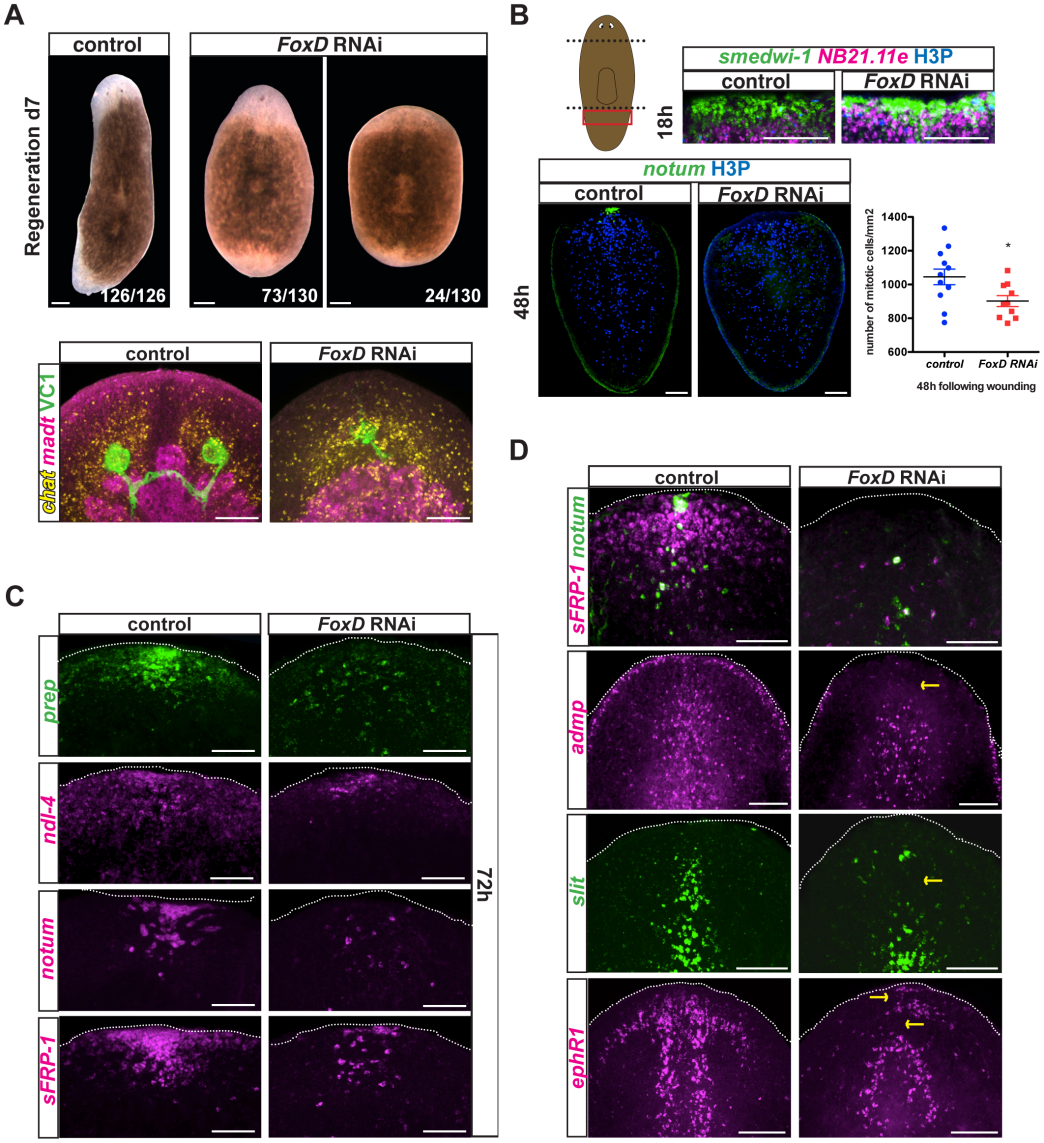
*FoxD(RNAi)* animals display abnormal anterior pole, head patterning and midline gene expression. (**A**) Upper panels: animals injected with *FoxD* dsRNA regenerated normally sized anterior blastemas with one eye (73/130) or smaller blastemas with no eyes (24/130) seven days following amputation. Scale bars, 200 µm. Lower panels: double FISH and immunostainings using anti-ARRESTIN (VC1) antibody in regenerating *FoxD* dsRNA injected animals. Defects in head regeneration of *FoxD(RNAi)* animals were observed; probes used: brain, *chat* (7/7 animals showed a medially collapsed or a very small brain); intestine, *mat* (3/7 animals showed some regeneration defect in the anterior blastema). Images shown are maximal intensity projections. Scale bars, 100 µm. Anterior is up, dorsal view. (**B**) Upper panels: double FISH (*smedwi-1* in green, *NB.21.11e* in magenta) and immunostaining with anti-phospho histone 3 (H3P in blue) of tail fragments fixed at 18 hours following amputation of eight weeks RNAi fed animals. Red box in cartoon on the left shows the area imaged. Lower panels: FISH (*notum*, green) and immunostaining with anti-H3P (blue) were performed at 48 hours following amputation in regenerating tail fragments of eight weeks RNAi fed animals. Anterior is up, dorsal view. Images shown are maximal intensity projections. Scale bars, 100 µm. Numbers of mitotic cells were counted and normalized by the tail area (mm^2^) and analyzed using a Student-*t*-test analysis; *p<0.05, n>10. (**C**) *FoxD* dsRNA injected animals displayed defects in anterior pole gene and PCG expression at 72 hours following amputation (*sFRP-1*, 5/9; *ndl-4*, 3/4; *notum*, 10/11; *prep*, 5/6). Dotted white line marks the approximate animal edge. Images shown are maximal intensity projections. Anterior is up, dorsal view. Scale bars, 50 µm. (**D**) Single or double FISH in day seven regenerating dsRNA injected animals. Defective expression of the anterior pole gene *notum* (8/20 no expression, 10/20 decreased expression) and *sFRP-1* (9/18 no expression, 7/18 reduced expression) is observed in *FoxD(RNAi)* animals. Reduced expression of the midline genes *slit* (6/11 severe defect, 2/11 mild defect), *admp* (8/10 reduced expression), and *ephrin receptor 1, ephR1* (7/14 severe reduction, 3/14 mild reduction) was observed in *FoxD(RNAi)* animals. Yellow arrows point to missing or aberrant expression. Images shown are maximal intensity projections. Anterior is up, dorsal view for all panels except for *admp* and *slit* FISH. Scale bars, 100 µm.

*FoxD(RNAi)* animals displayed defective regeneration, with variable blastema size and heads that regenerated either one or no eyes (Fig. 5A). *FoxD(RNAi)* head blastemas had abnormal anatomy, with medial collapse of cephalic ganglia (labeled with an RNA probe to *choline acetyltransferase* (*chat*) [42]), one or no eyes (detected with an anti-ARRESTIN antibody (VC1) [43]), and a slightly abnormal anterior intestine morphology (labeled with an RNA probe to *mat* [1]) (Fig. 5A). Tail fragments regenerated pharynges (Fig. S4B), demonstrating that some missing tissues can regenerate in *FoxD(RNAi)* animals. Moreover, intestinal branches were normally regenerated in *FoxD(RNAi)* tail blastemas (Fig. S4C), indicating that *FoxD* has a largely specific role in anterior regeneration. Parasagittal thin fragments of *FoxD(RNAi)* animals also regenerated pharynges (Fig. S6B), further demonstrating that neoblasts can replace missing tissues. However, most of these fragments regenerated only one eye, showed slightly reduced expression of the anterior PCG *sFRP1*, and regenerated asymmetric cephalic ganglia (Fig. S6B).

Blastema size abnormalities in *FoxD(RNAi)* animals did not appear to be an overt consequence of a neoblast maintenance defect, because normal mitotic cell numbers were present in *FoxD(RNAi)* animals at the time of the amputation (0 hours following wounding, Fig. S4A). Following *FoxD* RNAi, neoblast (*smedwi-1*-expressing cells) proliferation (6 hours post-amputation, Fig. S4A) and migration (18 hours post-amputation, Fig. 5B, upper panel) in response to wounding were normal. At 48 hours following wounding, neoblasts of regenerating *FoxD(RNAi)* tail, but not trunk, fragments displayed slightly reduced neoblast proliferation (Fig. 5B and Fig. S4A). Reduced proliferation in *FoxD(RNAi)* tail fragments persisted five days following wounding (Fig. S4A).

*FoxD(RNAi)* animals have reduced numbers of *follistatin*-expressing cells at the anterior pole [22]. At 48 hours following wounding *notum^+^* and *notum^+^ follistatin^+^* cells were reduced from regenerating *FoxD(RNAi)* anterior blastemas (Fig. 5B and Fig. S5A). Furthermore, neoblasts expressing *notum* and *prep* were also fewer or absent in *FoxD(RNAi)* animals (Fig. S7), suggesting that *FoxD* is required for neoblast specification into anterior pole cell progenitors. These observations suggest a role for *FoxD* in anterior pole regeneration by specifying pole progenitors.

At 72 hours following head amputation, we observed significantly lower or complete absence in expression of the anterior PCGs *prep*, *ndl-4*, and *sFRP-1*, and decreased or no *notum*-coalesced cells in *FoxD(RNAi)* animals (Fig. 5C). After seven days of regeneration, *FoxD(RNAi)* anterior blastemas also showed severe defects in the expression of *sFRP-1* and *ndl-4*, as well as the pole-restricted gene *notum* (Fig. 5D and Fig. S5B). Together, these results establish a role for *FoxD* and the anterior pole in head patterning during regeneration.

**Figure 6:**
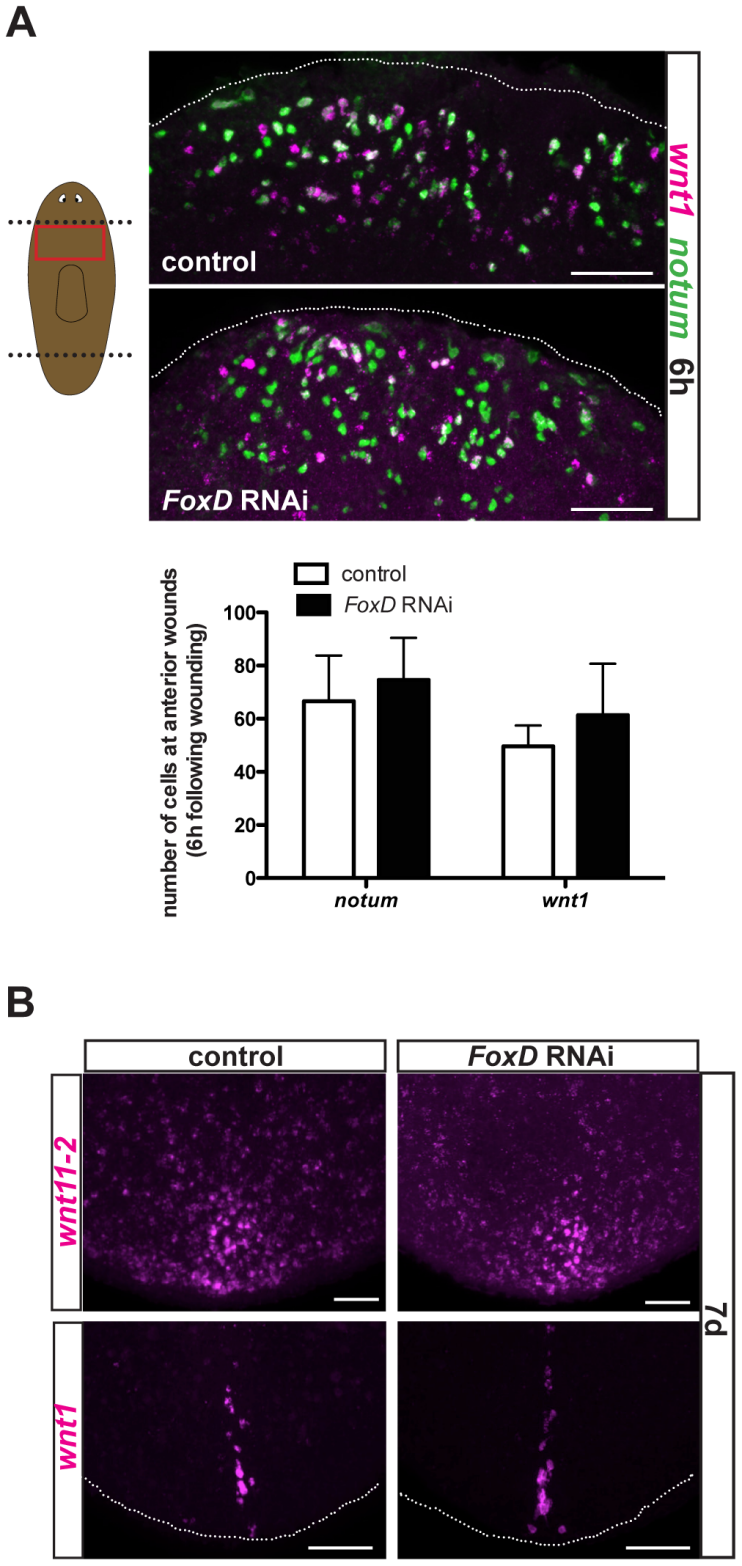
*FoxD(RNAi)* animals have normal expression of wound-induced genes and posterior genes. (**A**) Double FISH using *notum* (green) and *wnt1* (magenta) in trunk fragments of control and *FoxD* dsRNAi injected animals six hours following amputation. Red box in cartoon on the left shows the area imaged. Graph shows total number of cells expressing *notum*, or *wnt1* in the different RNAi conditions. Data are shown as mean± SD, n>5 animals per group. Anterior is up, ventral view. (**B**) Posterior blastemas at day seven of regeneration of *FoxD* dsRNA injected animals are shown; expression of posterior patterning gene *wnt11-2* (17/17) and the posterior pole gene *wnt1* (14/14) was normal in *FoxD(RNAi)* animals. Anterior is up, dorsal view. For all panels, images shown are maximal intensity projections. Scale bars, 50 µm.

*FoxD(RNAi)* animals had normal numbers of *notum*- and *wnt1*-expressing cells at six hours following wounding (Fig. 6A), indicating that *FoxD* does not prevent the wound-induced phase of *notum* expression during the specification of head regeneration. Furthermore, wound-induced expression of *follistatin* [22], [23] was normal in *FoxD(RNAi)* animals (Fig. S5A). We also did not observe ectopic expression of posterior markers (*wnt11-2* and *wnt1*) in regenerating anterior blastemas of *FoxD(RNAi)* animals (Fig. S4D), demonstrating that the choice to regenerate a head instead of a tail (AP regeneration polarity) was not detectably affected. In addition, we observed normal expression of posterior markers (*wnt11-2* and *wnt1*) in regenerating posterior blastemas, indicating a specific role for *FoxD* in anterior regeneration (Fig. 6B).

### *pbx* and *prep* are required for *FoxD* expression during anterior pole formation

**Figure 7:**
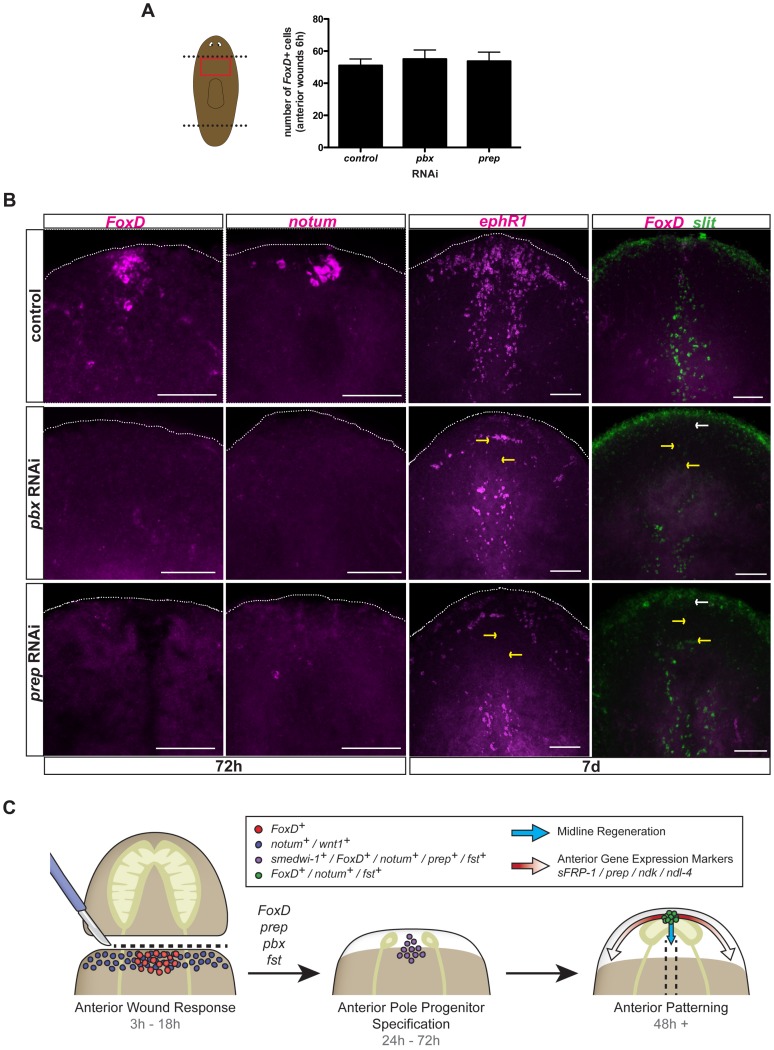
*pbx* and *prep* are required for *FoxD* pole expression, and anterior pole and midline regeneration. (**A**) Single FISH for *FoxD* in trunk fragments of control, *pbx*, and *prep* RNAi animals six hours following amputation. Graph shows total number of cells expressing *FoxD* in the different RNAi conditions. Data are shown as mean ± SD, n>9 animals per group. (**B**) Single or double FISH in regenerating *pbx* and *prep* RNAi animals. No *FoxD* or *notum* expression in anterior blastemas at 72 hours (n>5 animals per condition) and no *FoxD* expression at seven days (n>6 animals per panel) were observed in *pbx* and *prep* RNAi animals. No extension of *slit* expression to the anterior pole (5/5 animals), and defective expression of *ephrin receptor 1, ephR1* (n>4 animals per group) was observed in both *pbx* and *prep* RNAi animals. Yellow arrows point to missing or aberrant expression of midline genes. White arrows point to absence of *FoxD* expression at the anterior pole. Dotted white line marks the approximate animal edge. Images shown are maximal intensity projections. Anterior is up, dorsal view except in *slit* panels. Scale bars, 100 µm. (**C**) Proposed model for anterior regeneration: Following head amputation, wound-induced genes, like *notum* and *wnt1* (blue dots) are expressed throughout the wound, whereas *FoxD* is induced in the ventral midline (red dots). Note that *wnt1* is also wound-induced at posterior-facing wounds and that the gene *follistatin* (*fst*) is also wound induced. Subsequently, specification of anterior pole progenitors occurs within neoblasts. *FoxD*, *prep*, *pbx*, and *fst* are required for this step. *fst* RNAi can cause a strong block in regeneration, but under weaker RNAi conditions early expression of *notum* in this time window was affected [22]. Cells co-expressing the neoblast marker *smedwi-1* and the anterior-pole markers *FoxD* or *notum*, or the anterior-patterning gene *prep* are found within the anterior midline (purple dots). In a third phase, once the anterior pole is formed (cells expressing *FoxD*, *notum*, and *fst*; green dots), this pole organizes pattern of head blastemas: the cephalic ganglia, anterior gene expression domains (red arrows), and the regeneration of the midline (blue arrows).

The TALE-homeodomain-encoding *pbx* and *prep* genes are required for regeneration and maintenance of anterior PCG expression, including for markers of the anterior pole [17], [19], [20]. Because *prep* is expressed broadly at the head tip and *pbx* in most cells of the regenerating head [17], [19], [20], it was not previously possible to determine whether poles promoted anterior PCG expression or vice versa. However, because *FoxD* expression is restricted to the regenerating pole, the *FoxD* RNAi phenotype described above suggests the pole is required for anterior PCG expression. This model predicts that *pbx* and *prep* might be required for *FoxD* expression. Wound-induced *FoxD* expression at six hours following injury was normal in both *pbx* and *prep* RNAi animals (Fig. 7A). Similarly, other wound-induced genes (*notum* and *wnt1*) are expressed normally in *pbx(RNAi)* animals [19], [20], indicating that *pbx* and *prep* act downstream of wound-induced expression of these genes. By contrast, anterior pole-specific expression of *FoxD* and *notum* was completely absent in both *pbx* and *prep* RNAi animals 72 hours following head amputation (Fig. 7B). Because both *FoxD* and *notum* expression is induced in neoblasts at 72 hours following amputation of wild-type animals, the complete absence of expression of these two genes at this time point suggests a requirement for *pbx* and *prep* in the specification of neoblasts into anterior pole progenitors and that this defect might underlie the *pbx* and *prep* phenotypes.

### *FoxD* is required for midline regeneration

The restricted, wound-induced expression of *FoxD* in the midline raises the possibility of a regenerative connection between the midline and the anterior pole. To explore this possibility further, we analyzed the expression pattern of several midline genes, such as *slit*, *admp*, and *ephR1*, in *FoxD(RNAi)* animals. Indeed, these midline genes were not properly expressed in regenerating *FoxD(RNAi)* anterior blastemas (Fig. 5D). In addition, parasagittal thin pieces showed reduced slit expression at the anterior midline (Fig. S6B). By contrast, tail blastemas of transversely amputated *FoxD(RNAi)* animals displayed normal extension of *slit*-expressing cells to the posterior pole (Fig. S4C), further demonstrating the specific role of *FoxD* in the biology of the anterior pole.

To further investigate the possible connection of the regenerating anterior pole and the midline, we examined the midline-expressed *slit* and *ephR1* genes in *pbx* and *prep* RNAi animals, which are unable to regenerate an anterior pole (Fig. 7B and [19], [20]). *pbx(RNAi)* animals have been reported to have defective *slit* expression in anterior and posterior blastemas [19], [20]. As predicted, expression of both *slit* and *ephR1* was defective in anterior blastemas of *pbx* and *prep* RNAi animals seven days following head amputation (Fig. 7B). Moreover, the midline expression of *ephR1* was defective in all *ptc(RNAi)* animals showing intermediate phenotypes (cyclopia and headless; and therefore, reduced or absent anterior pole) at seven days following head amputation (Fig. S3D); because of the broad role of *ptc* it is unknown whether this defect is solely explained by a pole defect in *ptc(RNAi)* animals. *hh(RNAi)* animals, which can regenerate a normal anterior pole (Fig. S3B), showed proper expression of the *ephR1* gene in anterior blastemas at seven days following amputation (Fig. S3B). Intact *FoxD(RNAi)* animals undergoing long-term RNAi did not show an obvious phenotype (Fig. S6A); following transverse amputation of these animals, small blastemas with one or no eyes were regenerated, and the anterior pole was rarely formed or very reduced (Fig. S6C, D). In some of these amputated *FoxD(RNAi)* animals a small cluster of anterior pole cells were regenerated but offset from the midline (n = 5/19 offset, with 14/19 showing medial but severely reduced poles, Fig. S6D). Altogether, our results suggest a role for the anterior pole in organizing the regeneration of the new midline and patterning of the head.

## Discussion

The ends of embryos that will become the poles of the primary body axis can be involved in organizing embryonic tissue pattern in many animals. For example, *bicoid* mRNA in *Drosophila* is locally translated at the prospective anterior end of the oocyte and controls establishment of AP body pattern [44], [45]. PAR-3/PAR-6/aPKC-3 proteins localize to one side of the *C. elegans* zygote after fertilization, establishing the anterior embryo end [46]. In neurula-stage *Xenopus* embryos, Wnt genes regulate AP neurectoderm patterning and are active at the posterior, with Wnt inhibitory genes active at the anterior [47]. Early steps in development, such as asymmetric deposition of maternal determinants in oocytes or events triggered at the sperm entry site can be involved in establishment of polarized regions that impact primary axis pattern formation. In animals capable of whole-body regeneration, by contrast, the anterior and posterior poles must be formed *de novo* without reliance on events specific for embryogenesis. This problem exemplifies a general challenge faced by all regeneration paradigms: the establishment of pattern in an adult tissue context utilizing initial steps that can differ from the cues accessible to embryos. Planarian pole regeneration presents an ideal setting to explore the mechanistic basis for this process.

*FoxD* encodes a Forkhead-family transcription factor that functions during planarian regeneration to specify cells at a key signaling center occurring at the intersection of the anterior extreme and the midline. Transcription factors expressed regionally in embryos can promote establishment of signaling domains that control tissue pattern. A classic example is *goosecoid*, which encodes a homeodomain transcription factor required for expression of many patterning factors at Spemann's organizer in frog embryos [48]. In regeneration, how transcription factors might be suitably activated to organize regeneration of tissue pattern is poorly understood. The early and restricted midline expression of *FoxD* following wounding, the subsequent expression of *FoxD* at the anterior pole (which is located at the midline), and the anterior pole and midline regeneration phenotype in *FoxD*, *pbx*, and *prep* RNAi animals suggest that the anterior pole organizes many aspects of head regeneration. We propose the following model for head regeneration (Fig. 7C):

### (i) Anterior wound response

In the first phase, the anterior wound response (3–18 hours post-amputation), signaling mechanisms occurring in pre-existing muscle cells at wounds determine whether a new head is to be regenerated. Wound signaling triggers rapid (within ∼6 hours) expression of *wnt1* at all wounds [37]. Wnt signaling is selectively inhibited at anterior-facing wounds, involving activation of the *notum* gene [8]. The mechanism that leads to selectivity in *notum* activation is unknown. This first phase of head regeneration results in inhibition of Wnt signaling at anterior-facing wounds, whereas Wnt signaling is active at posterior-facing wounds. *FoxD* is activated at the midline of most wounds during this initial phase of wound-induced *wnt1* and *notum* expression, but our data do not indicate a role for this gene in the decision to regenerate a head-versus-tail.

### (ii) Anterior pole progenitor specification

If an anterior-facing wound is not juxtaposed by anterior tissue, a second phase of head regeneration involving anterior pole progenitor specification ensues. This phase (24–72 hours post-amputation) involves formation of a new anterior pole in an emerging blastema. The initial medial *FoxD* expression presents a candidate mechanism for establishing the location of pole regeneration–at the prior midline. This location of *FoxD* induction highlights the connection between the midline and pole regeneration as an important area for further investigation. We found that a small cluster of cells at the midline expressing *notum*, *follistatin*, and *FoxD* emerges from neoblasts in this second phase. Neoblasts can be specified to form eyes [48] or protonephridia [49] in regeneration, and here we demonstrate that some neoblasts near the forming anterior pole express *FoxD*, *notum*, and *prep*. We propose that neoblast induction of these genes near the midline at wounds represents an initial cellular step in formation of the cells of the new anterior pole.

### (iii) Anterior patterning

In a third phase of head regeneration (∼48 hours+ post-amputation), as the blastema grows substantially, pattern of the head blastema is established. *FoxD* RNAi severely reduced anterior pole regeneration. Whereas these *FoxD(RNAi)* anterior blastemas still displayed anterior character (such as the presence of brain cells), the blastemas lacked the normal tissue organization of wild-type head blastemas. *FoxD(RNAi)* blastemas also had defects in the expression of a number of anterior PCGs with broad anterior expression domains (e.g., *prep, sFRP-1*). This raises the possibility that the anterior pole is involved in establishing and maintaining more general anterior patterns of gene expression during this third phase of head regeneration. Consistent with this hypothesis, head tissue can also be regenerated in *pbx* or *prep* RNAi animals lacking poles, but establishment of anterior patterning gene expression domains is severely affected [17], [19], [20]. Finally, expression domains of genes expressed at the midline were aberrant in *FoxD(RNAi)* head blastemas. Together, these results suggest that the regenerating anterior pole promotes midline regeneration for proper bilateral patterning of the head and promotes establishment of gene expression domains for AP patterning of the head.

## Materials and Methods

### Animals and radiation treatment

Asexual *Schmidtea mediterranea* strain (CIW4) animals starved 7–14 days prior experiments were used. Animals were exposed to a 6,000 rads dose of radiation using a dual Gammacell-40 137 cesium source and amputated three days after irradiation. No vertebrate animals were used in this study, and usage of planarians (invertebrates) is unregulated.

### RNAi

Animals were injected with control (the *C. elegans* gene *unc-22*), *FoxD*, or *pbx* dsRNA. Animals were transversely amputated and trunk pieces injected within 1 hour post-amputation with dsRNA. A booster dsRNA injection of trunk pieces was performed the following day. This procedure (amputation, injection and booster injection) was performed a total of three times, every two to three days. Following the third cycle of amputation and injections, animals were scored for phenotype at 72 hours and seven days post amputation, fixed and analyzed with *in situ* hybridizations [50] and for many experiments involved azide quenching as described [50].

For RNAi by feeding experiments, dsRNA-expressing bacteria cultures were mixed with 70% liver solution in a 1∶300 ratio to culture volume. β-*catenin*, *APC*, and *prep* RNAi animals have been fed four times (days 0, 4, 7 and 11), and amputated at d12. 72 hours or seven days following amputation, animals were fixed and *in situ* hybridizations performed. *hh* and *ptc* RNAi animals were fed six times every three to four days. Long-term *FoxD(RNAi)* homeostasis experiments were performed by feeding the animals every three to four days during a ten week-period.

### Whole-mount *in situ* hybridizations and immunostainings

Animals were wounded and fixed at six hours following injury in 4% formaldehyde [50] and nitroblue tetrazolium/5-bromo-4-chloro-3-indolyl phosphate (NBT/BCIP) colorimetric whole-mount *in situ* hybridizations or fluorescence *in situ* hybridizations (FISH) were performed as described [50]. For immunostainings, animals were fixed as for *in situ* hybridizations and then treated as described [51]. A mouse anti-ARRESTIN antibody was kindly provided by Kiyokazu Agata and used in a 1∶5,000 dilution, and an anti-mouse-Alexa conjugated antibody was used in a 1∶500 dilution. For the neoblast wound response assay, RNAi animals were fed during the course of eight weeks every three to four days, then were transversely amputated and trunk or tail fragments were fixed at 0, 6, 18, 48, and 120 hours following wounding. Animals were immunostained using a rabbit anti-phospho histone 3 antibody and an anti-rabbit HRP in a 1∶100 dilution as previously described [52]. Fluorescent images were taken with a Zeiss LSM700 Confocal Microscope. Light images were taken with a Zeiss Discovery Microscope.

## Supporting Information

Figure S1 Phylogenetic analysis of SMED-FoxD. 93 Fox proteins from diverse organisms were aligned using ClustalW with default settings and trimmed with Gblocks. Maximum likelihood analyses were run using PhyML with 1,000 bootstrap replicates, the WAG model of amino acid substitution, four substitution rate categories and the proportion of invariable sites estimated from the dataset. The result provides strong support for the SMED-FoxD clade (705 out of 1,000, highlighted in red) to be a class D member of the Forkhead transcription family. All ML bootstrap values are shown. Hs, *Homo sapiens*; Mm, *Mus musculus*; Dm, *Drosophila melanogaster*; Smed, *Schmidtea mediterranea*; Xl, *Xenopus laevis*; Sd; *Suberites domuncula*; Bf, *Branchiostoma floridae*; Ci, *Ciona intestinalis*; Hv, *Hydra vulgaris*; Nv, *Nematostella vectensis*; Dj, *Dugesia japonica*; Cs, *Ciona selvatgi*; Ml, *Mnemiopsis leidyi*.
(TIF)

Figure S2 *FoxD* is partially co-expressed with *notum* and *wnt1* following wounding and localizes to the anterior-facing wounds 72 hours following amputation. (**A**) FISH using *FoxD* (magenta) in a wild-type trunk piece fixed 72 hours post-amputation. Cartoon on the left shows the area imaged. Yellow arrow points to anterior expression of *FoxD*. Image shown is a maximal intensity projection. Image is representative of results seen in >20 animals. Anterior is up, dorsal view. Scale bar, 100 µm. **(B**) Double FISH using *FoxD* (magenta) and *notum* or *wnt1* (green) RNA probes in wild-type animals at six hours following amputation. Cartoon on the left shows the area imaged. Dotted white line depicts the wound boundary. Images shown are maximal intensity projections. Images are representative of results seen in >8 animals. Yellow arrows point to co-expression of *FoxD* and *notum* or *wnt1*. Percentages (mean ± SD) of *FoxD* cells co-expressing *notum* was 58.4±9.4% (n = 270 *FoxD^+^* cells examined) and *FoxD* co-expression with *wnt1* was 50±17% (n = 258 *FoxD^+^* cells examined). Anterior is up, ventral view. Scale bar, 100 µm.
(TIF)

Figure S3 Hh signaling impacts wound-induced *FoxD* expression and the regeneration of the anterior pole and midline. (**A**) Graphs show numbers of cells expressing *FoxD* or *wnt1* in different RNAi conditions. Data are shown as means ± SD, and analyzed using a one-way ANOVA test; *p<0.05; **p<0.01, ***p<0.001, n>10 animals per RNAi condition. (**B**) Single FISH using *FoxD* (upper panels) or *ephR1* (lower panels) RNA probes in seven days regenerating RNAi fed animals. Images are representative of results seen in >5 animals per condition. (**C**) Single FISH using the RNA probe *slit* (magenta) in RNAi fed animals at six hours following amputation. Images are representative of results seen in >8 animals per condition. (**D**) Single FISH using the RNA probes *notum*, *sFRP1* and *ephR1* (magenta) in RNAi fed animals at 72 hours or seven days following head amputation. Reduced expression of *notum* and *sFRP1* was observed in 8/8 cyclopic and in 3/3 headless *ptc(RNAi)* animals. Defective expression of *ephR1* was observed in 8/8 *ptc(RNAi)* animals. Dotted white line depicts the animal edge. All images shown are maximal intensity projections. For all panels, anterior is up, dorsal view. Scale bars, 100 µm.
(TIF)

Figure S4 *FoxD(RNAi)* animals have a normal neoblast wound response, normal polarity, and regenerate the pharynx and posterior tissues. (**A**) Graphs show numbers of mitotic cells counted and normalized by the tail or trunk area (mm^2^). Data are shown as means± SD and analyzed using a Student-*t*-test analysis; *p<0.05, n>5 fragments per time point. (**B**) Single FISH using the *mhc* RNA probe (magenta) in day seven regenerating tail pieces of eight week RNAi fed animals. These fragments did not have a pharynx before regeneration. Red box in cartoon on the left shows the tail fragment before regeneration. Images are representative of results seen in >4 animals per panel. (**C**) Single FISH using *slit* (green) and *mat* (magenta) RNA probes at seven days following transverse amputation of dsRNA injected animals. Images are representative of results seen in >7 animals per panel. (**D**) Double FISH using *sFRP-1* (magenta) and *wnt11-2* (green) RNA probes in regenerating trunk pieces of control and *FoxD(RNAi)* animals at 72 hours following transverse amputation. Red box in cartoon on the left shows the area imaged. Dotted white line depicts the animal edge. Images are representative of results seen in >4 animals per panel. All images shown are maximal intensity projections. Anterior is up, dorsal view. Scale bars, 100 µm.
(TIF)

Figure S5 *FoxD(RNAi)* animals regenerate a defective anterior pole. (**A**) Double FISH in control and *FoxD* RNAi fed animals using *follistatin* (magenta) and *notum* (green) RNA probes at 48 hours post-amputation. Red box in cartoon on the left shows the area imaged. Dotted white boxes are shown at higher magnification on the right panels. Yellow arrows point to cells found at the anterior midline expressing both *notum* and *follistatin*. Dotted white line depicts the animal edge. Images are representative of results seen in >5 animals. Scale bar in left panels, 100 µm and in right panels, 50 µm. Graph shows number of cells expressing both *notum* and *follistatin* at the forming anterior pole in different RNAi conditions. Data are shown as means ± SEM, and analyzed using a Student t-test; *p<0.05. (**B**) Single FISH using *ndl-4* RNA probe (magenta) at day seven following head amputation of dsRNA-injected animals. Images are representative of results seen in >5 animals per panel. Scale bar, 100 µm. All images shown are maximal intensity projections. Anterior, up, dorsal view.
(TIF)

Figure S6 Regeneration of *FoxD(RNAi)* animals following long term homeostasis RNAi feedings is defective. (**A**) *FoxD(RNAi)* animals appeared normal following ten weeks of RNAi feedings (33/33). Following this period of time, animals were parasagittally or transversely amputated. Red box in cartoons on the right shows the fragments imaged in (B and C) before regeneration. Anterior is up, dorsal view. Scale bars, 200 µm. (**B**) Following parasagittal amputations, *FoxD(RNAi)* thin fragments regenerated one eye (10/14) or no eyes (3/14) (left panels). White arrows point to eyes. FISH of thin fragments at day 12 following parasagittal amputation using the *sFRP1* (magenta) RNA probe and nuclear counterstaining using DAPI. Red arrows point to asymmetric regeneration of the cephalic ganglia (5/5 animals) (middle panels). FISH using *slit* (magenta) RNA probe. Yellow arrows point to decreased number of *slit*-expressing cells. White arrows point to pharynx regeneration. Images are representative of results seen in >4 animals per panel. Anterior is up. Scale bars, 200 µm. (**C**) Regeneration of transversely amputated *FoxD(RNAi)* animals following long-term RNAi (homeostasis experiments) resulted in a stronger blastema phenotype. 10/19 animals showed small blastemas and 6/19 animals regenerated one eye. Anterior is up, dorsal view. Scale bars, 200 µm. (**D**) FISH of animals in (C) using *sFRP1* (green) and *notum* (magenta) RNA probes. 7/19 animals showed no expression of *notum* or *sFRP1*, 7/19 animals had reduced numbers of *notum*- and *sFRP1*-expressing cells, and in 5/19 animals had few numbers of *notum*-expressing cells offset from the midline. White dotted line depicts the animal edge. Yellow dotted line depicts the estimate midline. Images shown are maximal intensity projections. Anterior is up. Scale bars, 100 µm.
(TIF)

Figure S7 *FoxD(RNAi)* animals have few anterior pole progenitors. Double FISH using the RNA probes *smedwi-1* (green) and *prep* or *notum* (magenta) in dsRNA-injected animals at 72 hours following amputation. (**A**) Control animals displayed double-expressing *smedwi-1* ^+^ */prep* ^+^ and *smedwi-1* ^+^/*notum* ^+^ cells. (**B**) *FoxD(RNAi)* animals showed reduced numbers of double-expressing *smedwi-1* ^+^/*prep* ^+^ cells (no expression of *prep* n = 3/7, severe reduction n = 4/7) and *smedwi-1* ^+^/*notum* ^+^ cells (no expression of *notum* n = 3/5, severe reduction n = 2/5). Images are representative of results seen in >5 animals per panel. Higher magnification (63×) example images are also shown. Yellow arrows point to double-positive cells for the markers analyzed. White dotted line depicts the animal edge. Images shown are maximal intensity projections. Anterior is up, dorsal view. Scale bars, 100 µm. Scale bars of inset, 10 µm.
(TIF)
