# Supplementary figures and images for: A forkhead Transcription Factor Is Wound-Induced at the Planarian Midline and Required for Anterior Pole Regeneration
Source: PLoS Genet. 2014 Jan 9;10(1):e1003999. doi: 10.1371/journal.pgen.1003999 (PMC3886891; doi:10.1371/journal.pgen.1003999)

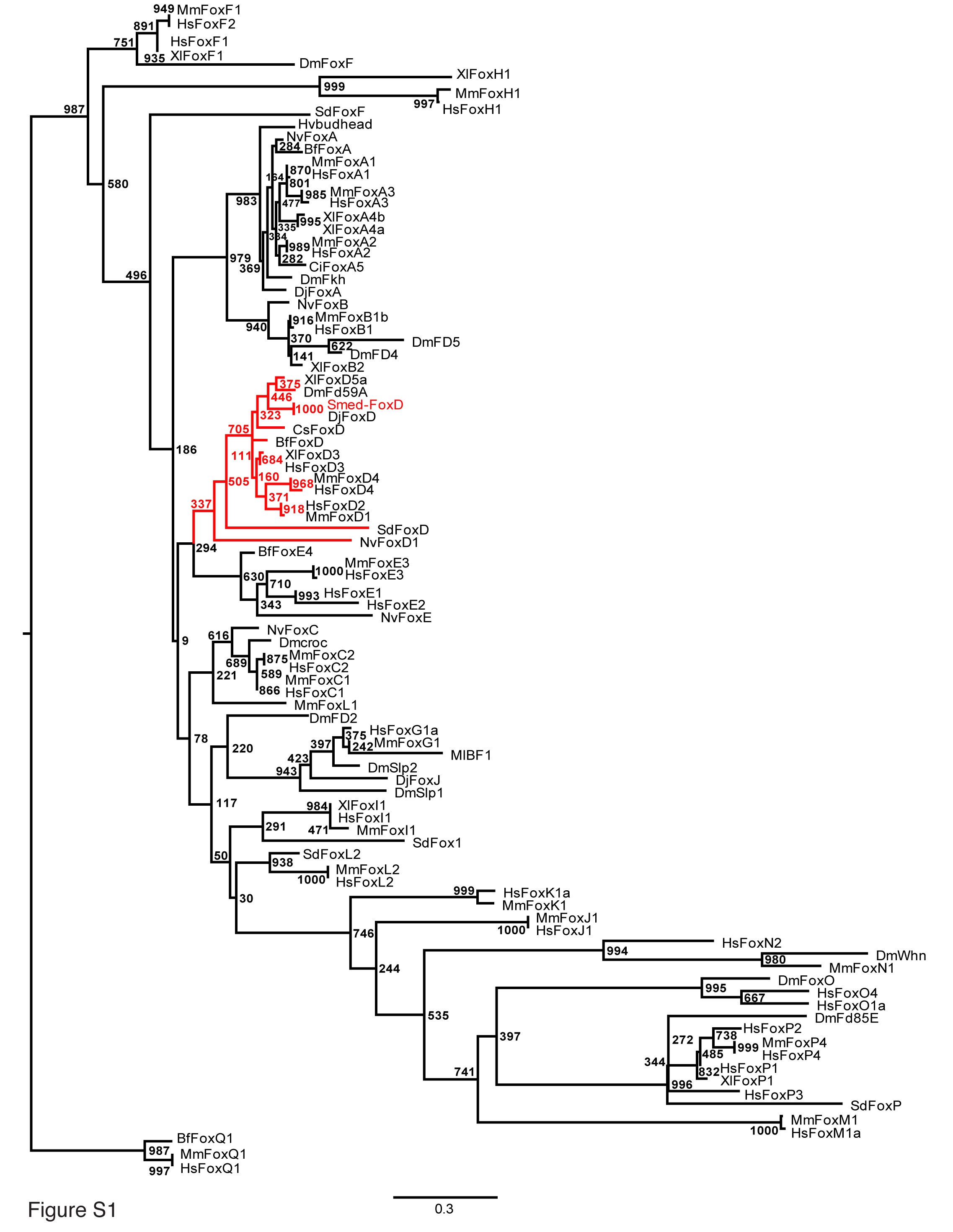

Supplement: Figure S1 — Phylogenetic analysis of SMED-FoxD. 93 Fox proteins from diverse organisms were aligned using ClustalW with default settings and trimmed with Gblocks. Maximum likelihood analyses were run using PhyML with 1,000 bootstrap replicates, the WAG model of amino acid substitution, four substitution rate categories and the proportion of invariable sites estimated from the dataset. The result provides strong support for the SMED-FoxD clade (705 out of 1,000, highlighted in red) to be a class D member of the Forkhead transcription family. All ML bootstrap values are shown. Hs, Homo sapiens; Mm, Mus musculus; Dm, Drosophila melanogaster; Smed, Schmidtea mediterranea; Xl, Xenopus laevis; Sd; Suberites domuncula; Bf, Branchiostoma floridae; Ci, Ciona intestinalis; Hv, Hydra vulgaris; Nv, Nematostella vectensis; Dj, Dugesia japonica; Cs, Ciona selvatgi; Ml, Mnemiopsis leidyi. (TIF) [file pgen.1003999.s001.tif]

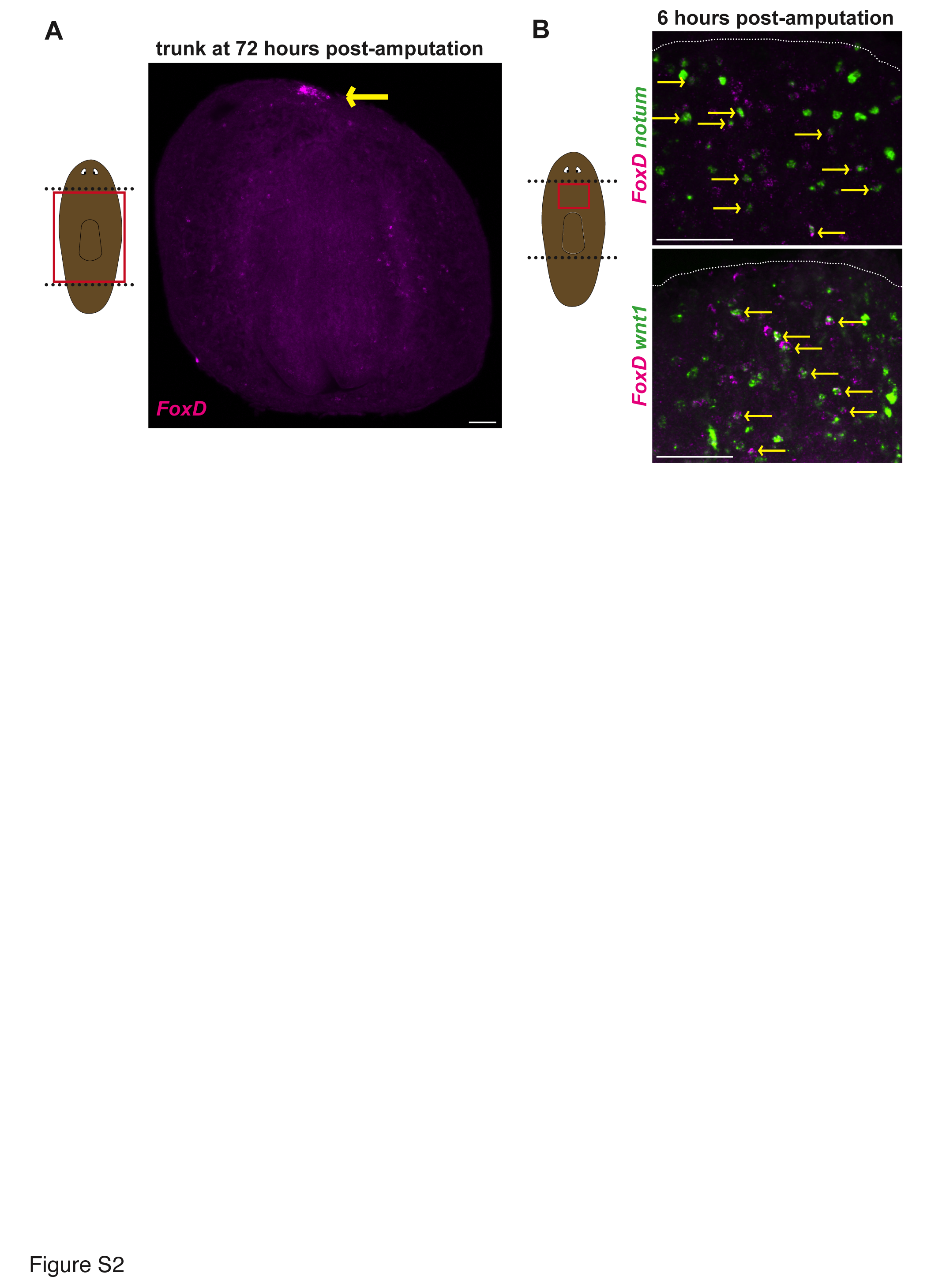

Supplement: Figure S2 — FoxD is partially co-expressed with notum and wnt1 following wounding and localizes to the anterior-facing wounds 72 hours following amputation. (A) FISH using FoxD (magenta) in a wild-type trunk piece fixed 72 hours post-amputation. Cartoon on the left shows the area imaged. Yellow arrow points to anterior expression of FoxD. Image shown is a maximal intensity projection. Image is representative of results seen in >20 animals. Anterior is up, dorsal view. Scale bar, 100 µm. (B) Double FISH using FoxD (magenta) and notum or wnt1 (green) RNA probes in wild-type animals at six hours following amputation. Cartoon on the left shows the area imaged. Dotted white line depicts the wound boundary. Images shown are maximal intensity projections. Images are representative of results seen in >8 animals. Yellow arrows point to co-expression of FoxD and notum or wnt1. Percentages (mean ± SD) of FoxD cells co-expressing notum was 58.4±9.4% (n = 270 FoxD+ cells examined) and FoxD co-expression with wnt1 was 50±17% (n = 258 FoxD+ cells examined). Anterior is up, ventral view. Scale bar, 100 µm. (TIF) [file pgen.1003999.s002.tif]

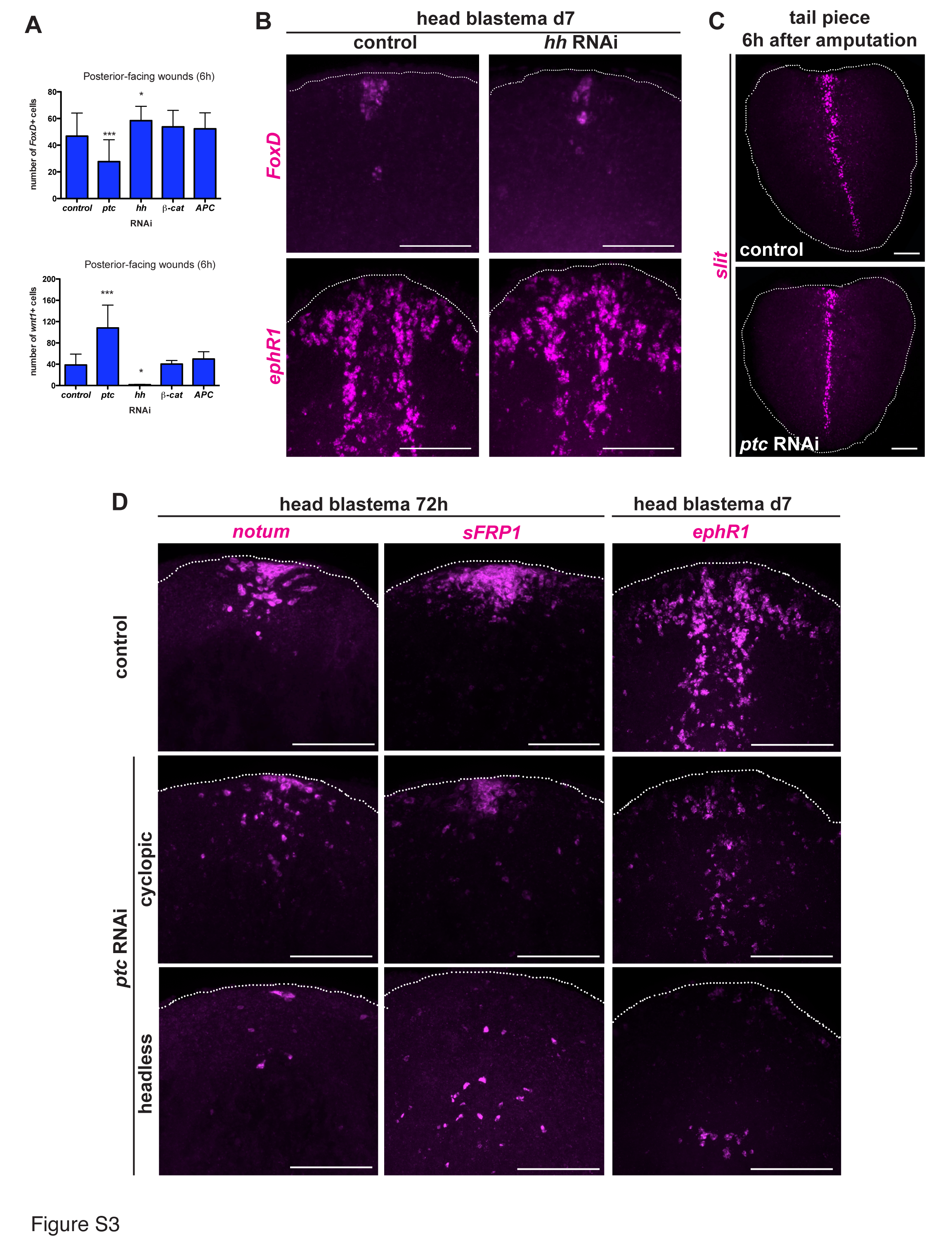

Supplement: Figure S3 — Hh signaling impacts wound-induced FoxD expression and the regeneration of the anterior pole and midline. (A) Graphs show numbers of cells expressing FoxD or wnt1 in different RNAi conditions. Data are shown as means ± SD, and analyzed using a one-way ANOVA test; *p<0.05; **p<0.01, ***p<0.001, n>10 animals per RNAi condition. (B) Single FISH using FoxD (upper panels) or ephR1 (lower panels) RNA probes in seven days regenerating RNAi fed animals. Images are representative of results seen in >5 animals per condition. (C) Single FISH using the RNA probe slit (magenta) in RNAi fed animals at six hours following amputation. Images are representative of results seen in >8 animals per condition. (D) Single FISH using the RNA probes notum, sFRP1 and ephR1 (magenta) in RNAi fed animals at 72 hours or seven days following head amputation. Reduced expression of notum and sFRP1 was observed in 8/8 cyclopic and in 3/3 headless ptc(RNAi) animals. Defective expression of ephR1 was observed in 8/8 ptc(RNAi) animals. Dotted white line depicts the animal edge. All images shown are maximal intensity projections. For all panels, anterior is up, dorsal view. Scale bars, 100 µm. (TIF) [file pgen.1003999.s003.tif]

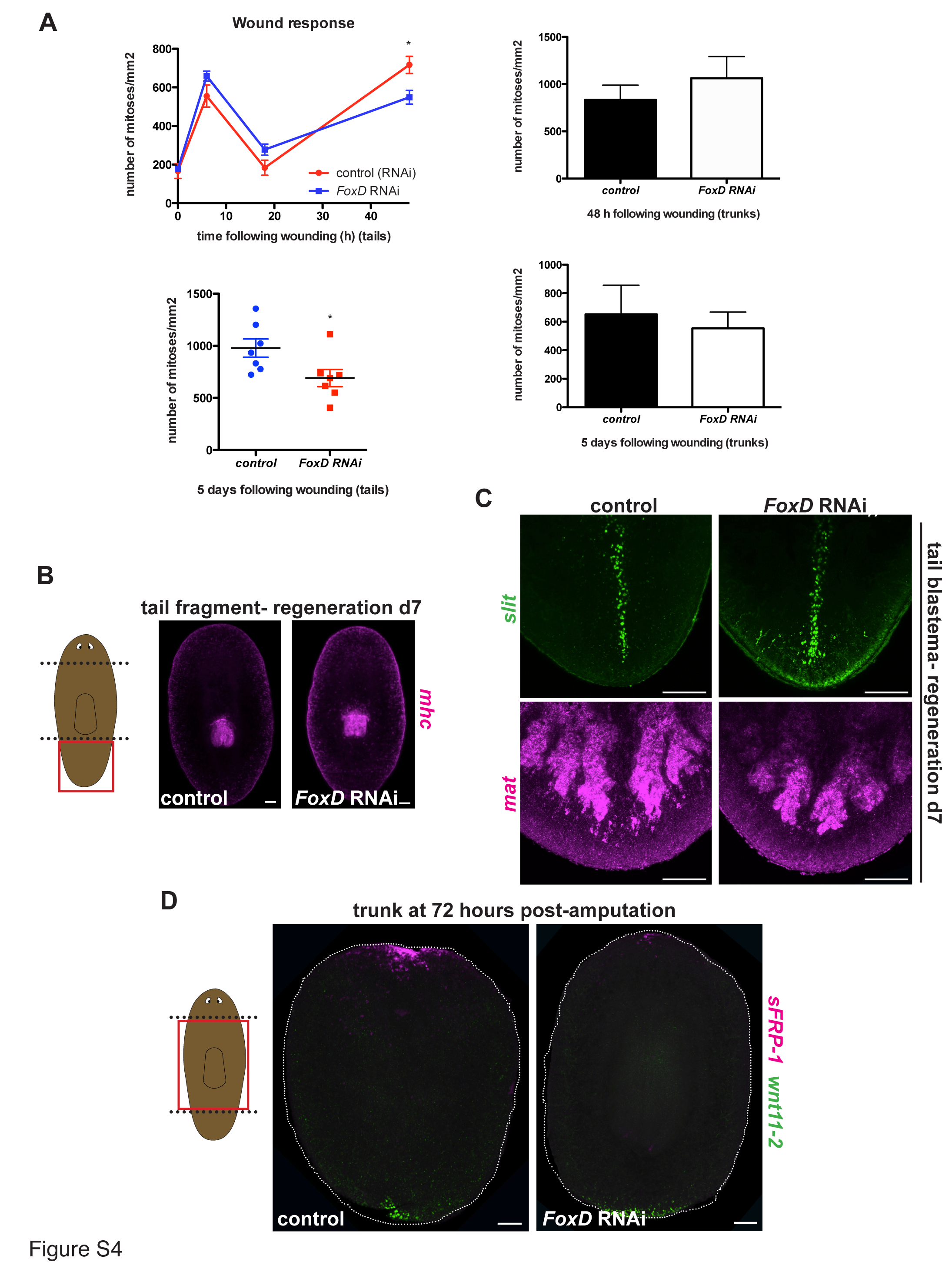

Supplement: Figure S4 — FoxD(RNAi) animals have a normal neoblast wound response, normal polarity, and regenerate the pharynx and posterior tissues. (A) Graphs show numbers of mitotic cells counted and normalized by the tail or trunk area (mm2). Data are shown as means± SD and analyzed using a Student-t-test analysis; *p<0.05, n>5 fragments per time point. (B) Single FISH using the mhc RNA probe (magenta) in day seven regenerating tail pieces of eight week RNAi fed animals. These fragments did not have a pharynx before regeneration. Red box in cartoon on the left shows the tail fragment before regeneration. Images are representative of results seen in >4 animals per panel. (C) Single FISH using slit (green) and mat (magenta) RNA probes at seven days following transverse amputation of dsRNA injected animals. Images are representative of results seen in >7 animals per panel. (D) Double FISH using sFRP-1 (magenta) and wnt11-2 (green) RNA probes in regenerating trunk pieces of control and FoxD(RNAi) animals at 72 hours following transverse amputation. Red box in cartoon on the left shows the area imaged. Dotted white line depicts the animal edge. Images are representative of results seen in >4 animals per panel. All images shown are maximal intensity projections. Anterior is up, dorsal view. Scale bars, 100 µm. (TIF) [file pgen.1003999.s004.tif]

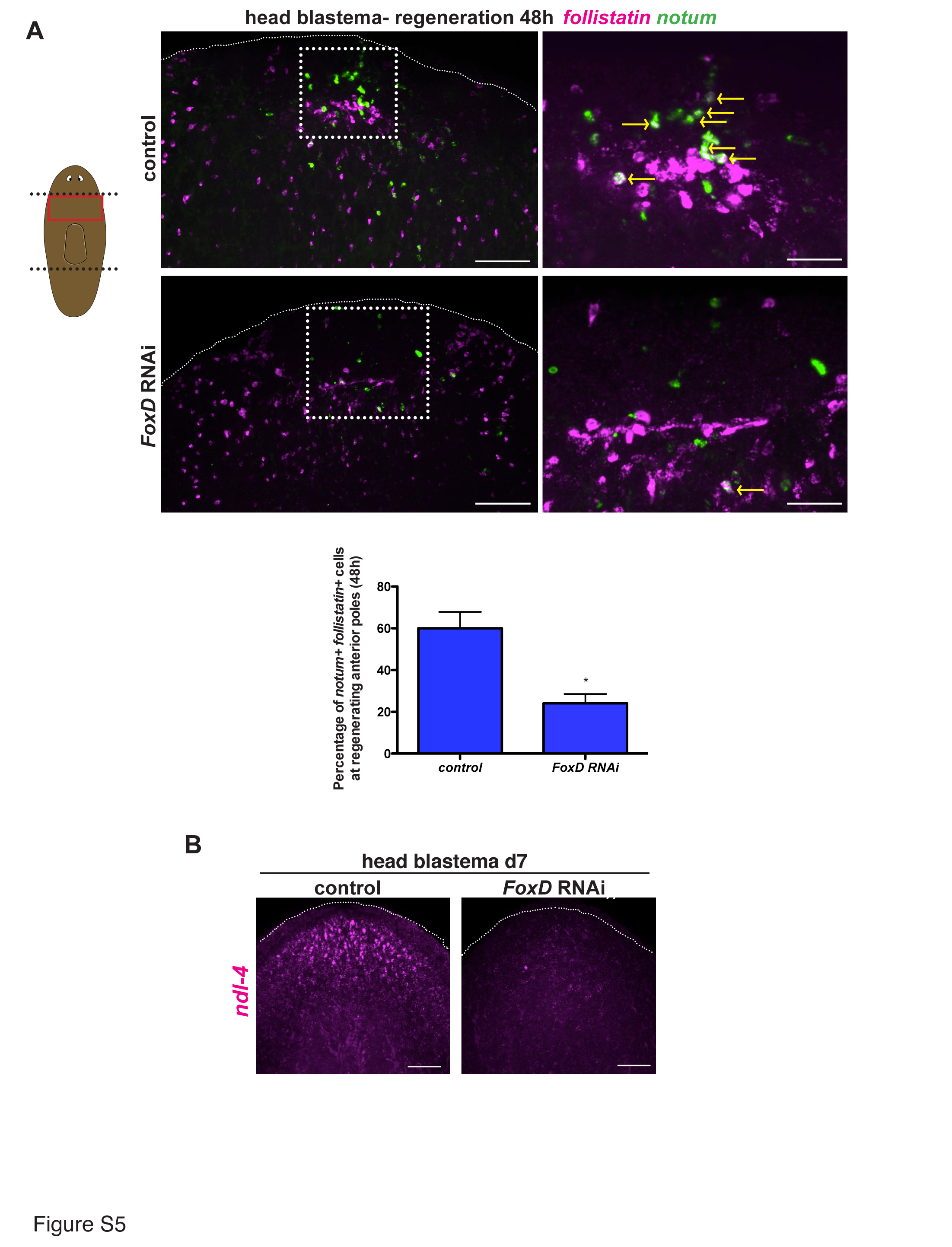

Supplement: Figure S5 — FoxD(RNAi) animals regenerate a defective anterior pole. (A) Double FISH in control and FoxD RNAi fed animals using follistatin (magenta) and notum (green) RNA probes at 48 hours post-amputation. Red box in cartoon on the left shows the area imaged. Dotted white boxes are shown at higher magnification on the right panels. Yellow arrows point to cells found at the anterior midline expressing both notum and follistatin. Dotted white line depicts the animal edge. Images are representative of results seen in >5 animals. Scale bar in left panels, 100 µm and in right panels, 50 µm. Graph shows number of cells expressing both notum and follistatin at the forming anterior pole in different RNAi conditions. Data are shown as means ± SEM, and analyzed using a Student t-test; *p<0.05. (B) Single FISH using ndl-4 RNA probe (magenta) at day seven following head amputation of dsRNA-injected animals. Images are representative of results seen in >5 animals per panel. Scale bar, 100 µm. All images shown are maximal intensity projections. Anterior, up, dorsal view. (TIF) [file pgen.1003999.s005.tif]

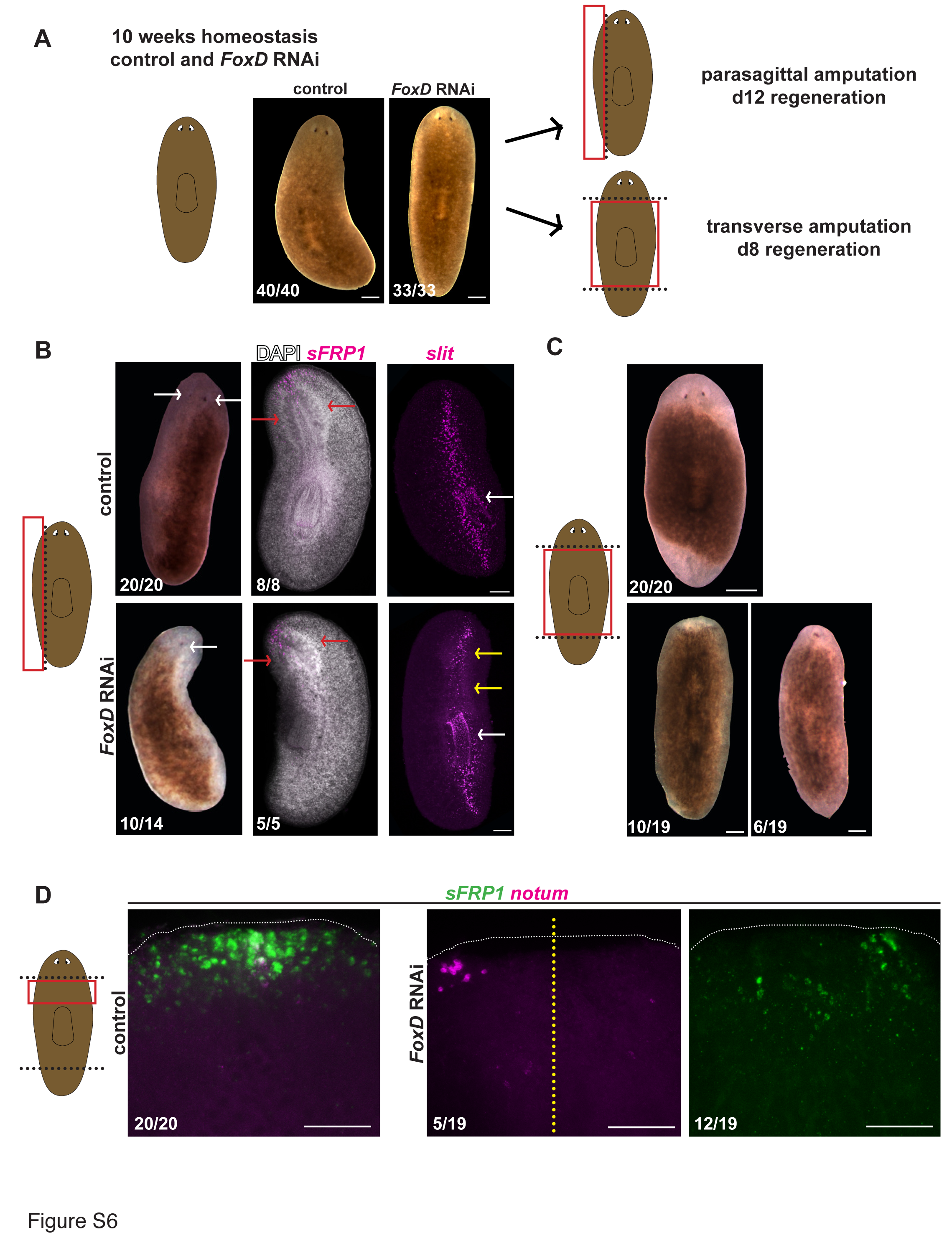

Supplement: Figure S6 — Regeneration of FoxD(RNAi) animals following long term homeostasis RNAi feedings is defective. (A) FoxD(RNAi) animals appeared normal following ten weeks of RNAi feedings (33/33). Following this period of time, animals were parasagittally or transversely amputated. Red box in cartoons on the right shows the fragments imaged in (B and C) before regeneration. Anterior is up, dorsal view. Scale bars, 200 µm. (B) Following parasagittal amputations, FoxD(RNAi) thin fragments regenerated one eye (10/14) or no eyes (3/14) (left panels). White arrows point to eyes. FISH of thin fragments at day 12 following parasagittal amputation using the sFRP1 (magenta) RNA probe and nuclear counterstaining using DAPI. Red arrows point to asymmetric regeneration of the cephalic ganglia (5/5 animals) (middle panels). FISH using slit (magenta) RNA probe. Yellow arrows point to decreased number of slit-expressing cells. White arrows point to pharynx regeneration. Images are representative of results seen in >4 animals per panel. Anterior is up. Scale bars, 200 µm. (C) Regeneration of transversely amputated FoxD(RNAi) animals following long-term RNAi (homeostasis experiments) resulted in a stronger blastema phenotype. 10/19 animals showed small blastemas and 6/19 animals regenerated one eye. Anterior is up, dorsal view. Scale bars, 200 µm. (D) FISH of animals in (C) using sFRP1 (green) and notum (magenta) RNA probes. 7/19 animals showed no expression of notum or sFRP1, 7/19 animals had reduced numbers of notum- and sFRP1-expressing cells, and in 5/19 animals had few numbers of notum-expressing cells offset from the midline. White dotted line depicts the animal edge. Yellow dotted line depicts the estimate midline. Images shown are maximal intensity projections. Anterior is up. Scale bars, 100 µm. (TIF) [file pgen.1003999.s006.tif]

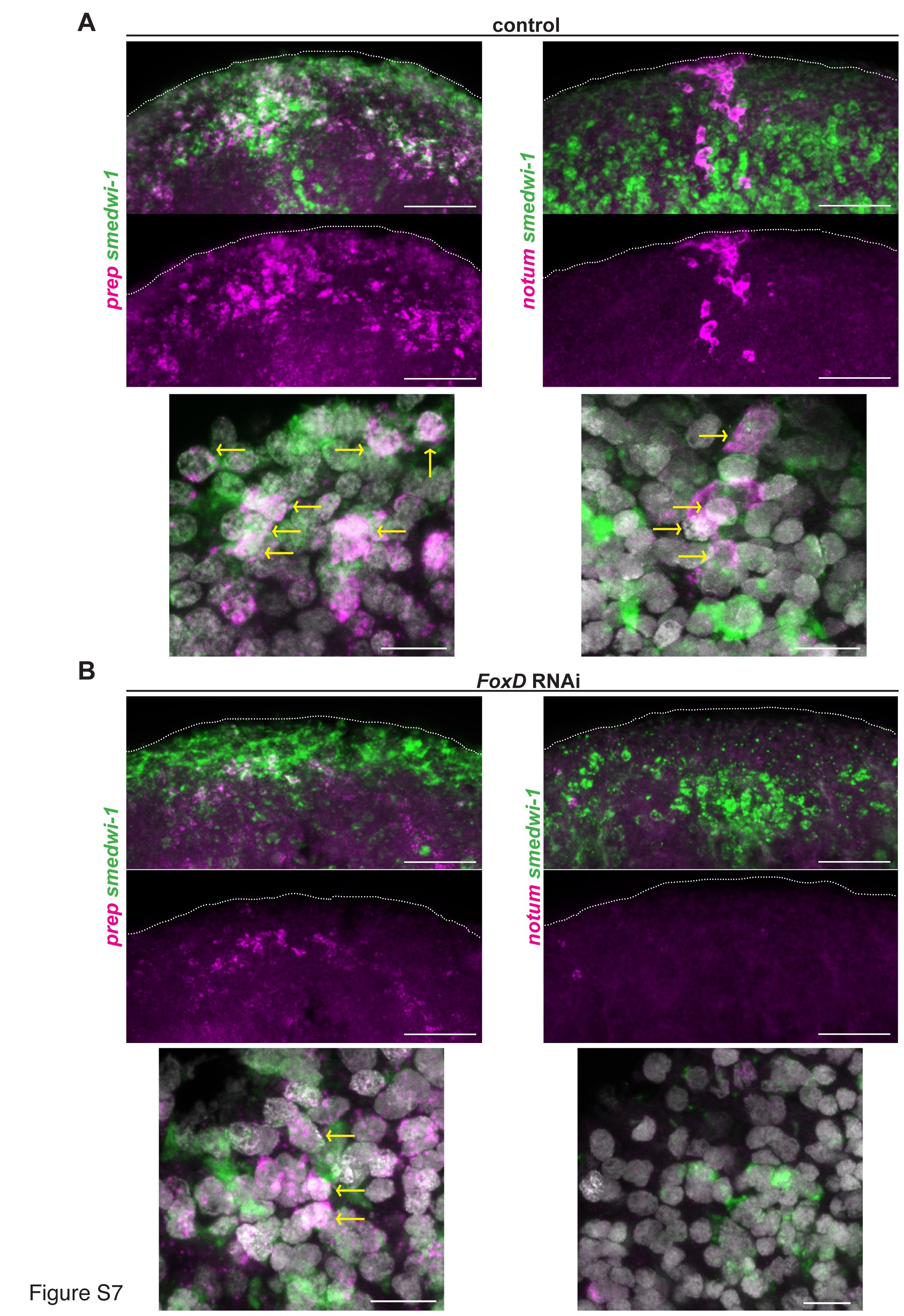

Supplement: Figure S7 — FoxD(RNAi) animals have few anterior pole progenitors. Double FISH using the RNA probes smedwi-1 (green) and prep or notum (magenta) in dsRNA-injected animals at 72 hours following amputation. (A) Control animals displayed double-expressing smedwi-1 + /prep + and smedwi-1 +/notum + cells. (B) FoxD(RNAi) animals showed reduced numbers of double-expressing smedwi-1 +/prep + cells (no expression of prep n = 3/7, severe reduction n = 4/7) and smedwi-1 +/notum + cells (no expression of notum n = 3/5, severe reduction n = 2/5). Images are representative of results seen in >5 animals per panel. Higher magnification (63×) example images are also shown. Yellow arrows point to double-positive cells for the markers analyzed. White dotted line depicts the animal edge. Images shown are maximal intensity projections. Anterior is up, dorsal view. Scale bars, 100 µm. Scale bars of inset, 10 µm. (TIF) [file pgen.1003999.s007.tif]
